# Genetic diversity and local adaption of alfalfa populations (*Medicago sativa* L.) under long-term grazing

**DOI:** 10.1038/s41598-023-28521-3

**Published:** 2023-01-30

**Authors:** Hu Wang, Bruce Coulman, Yuguang Bai, Bunyamin Tarˈan, Bill Biligetu

**Affiliations:** Department of Plant Sciences, College of Agriculture and Bioresources, University of Saskatchewan, Saskatoon, SK Canada

**Keywords:** Plant sciences, Plant stress responses, Abiotic

## Abstract

Genomic information on alfalfa adaptation to long-term grazing is useful for alfalfa genetic improvement. In this study, 14 alfalfa populations were collected from long-term grazing sites (> 25 years) across four soil zones in western Canada. Alfalfa cultivars released between 1926 and 1980 were used to compare degree of genetic variation of the 14 populations. Six agro-morphological and three nutritive value traits were evaluated from 2018 to 2020. The genotyping-by-sequencing (GBS) data of the alfalfa populations and environmental data were used for genotype-environment association (GEA). Both STRUCTURE and UPGMA based on 19,853 SNPs showed that the 14 alfalfa populations from long-term grazing sites had varying levels of parentages from alfalfa sub-species *Medicago sativa* and *M. falcata*. The linear regression of STRUCTURE membership probability on phenotypic data indicated genetic variations of forage dry matter yield, spring vigor and plant height were low, but genetic variations of regrowth, fall plant height, days to flower and crude protein were still high for the 14 alfalfa populations from long-term grazing sites. The GEA identified 31 SNPs associated with 13 candidate genes that were mainly associated with six environmental factors of. Candidate genes underlying environmental factors were associated with a variety of proteins, which were involved in plant responses to abiotic stresses, i.e., drought, cold and salinity-alkali stresses.

## Introduction

Cultivated alfalfa (*Medicago sativa* L.), a perennial auto-tetraploid (2n = 4x = 32) and cross-pollinated forage legume, is widely grown as forage due to its high yield and high adaptation in temperate regions of the world^1^. The cultivated alfalfa complex consists of three main sub-species: *M. sativa* subsp. *sativa*, *M. sativa* subsp. *falcata*, and *M. sativa* media^2^ with each sub-species displaying distinguishing agro-morphological traits^2,3^. Identification of genetic diversity and relationships within and among different alfalfa populations is of great importance for alfalfa genetic improvement. Especially, information on local adaption under long-term grazing is critical, as alfalfa stands are often affected by grazing^4^, and environmental conditions and their interactions^3,5^, which potentially results in genetic changes over time.

Long-term continuous grazing can deplete the alfalfa total non-structural carbohydrates (TNC) and N reserves in the root, and remove photosynthetically active leaf areas^6,7^, resulting in greater susceptibility to biotic and abiotic stresses, i.e., winter injuries and disease infections. A number of genomic studies demonstrated that plant genetic diversity decreased with increasing grazing intensities in several forage grasses, i.e., *Stipa grandis*^8^, *Elymus nutans* Griseb.^9^, blue grama (*Bouteloua gracilis*)^10^, and meadow fescue (*Festuca pratensis* Huds.)^11^. However, a few studies reported no evident genetic diversity change between grazed and ungrazed mountain rough fescue (*Festuca campestris* Rydb.) populations^12^, and Idaho fescue (*Festuca idahoensis* Elmer)^13^. The reason for no significant changes of genetic diversity in these two studies was that a high degree of gene flow occurred between the populations^13^. Recent studies indicated that the environmental heterogeneities alone can drive species to accumulate local adaptive loci^14,15^. Blanco-Pastor et al.^16^ identified 143 candidate genes of local adaptation primarily associated with environmental factors in diploid alfalfa populations. In western Canada, unique and highly variable environmental factors such as drought, heat, and extreme cold weather affect alfalfa survival and adaptation^17–21^. Lack of soil moisture during growing season is common in the regions, which can limit alfalfa persistence and productivity^3,22,23^.

In recent years, genome-wide single nucleotide polymorphisms (SNPs) by genotyping-by-sequencing (GBS) have been applied in the genetic diversity analysis of alfalfa including population structure analysis^24^. The GBS method makes it possible to generate high-density, genome-wide SNPs^25^. The abundant SNP markers are more informative than previously used molecular markers in the above-mentioned grass studies and have been successfully applied to identify the genetic structure and relationship in alfalfa breeding materials^26,27^. However, there are few genomic studies available in examining local adaptation of alfalfa populations under long-term grazing and varying environmental factors.

In this study, alfalfa populations with a minimum of 25 years of grazing history were collected from 14 ranch sites across four soil zones of Saskatchewan, Canada. It was hypothesized that: (1) there will be large phenotypic and genetic variations among the alfalfa populations adapted to long term grazing history; and (2) because of their stand age, the alfalfa populations from long-term grazing sites will be genetically related to one or more of commercial cultivars released from 1926 to 1980 in western Canada. However, each of the alfalfa populations will have unique adaptive loci due to long-term grazing effects and environmental heterogeneity at each ranch site. The objectives of this study were: (1) to identify the genetic relatedness of the 14 alfalfa populations with long-term grazing history inferred by 11 commercial alfalfa cultivars released from 1926 to 1980; and (2) to evaluate genetic and phenotypic variations of 14 alfalfa populations from long-term grazing sites.

## Results

### Population structure

**Figure 1:**
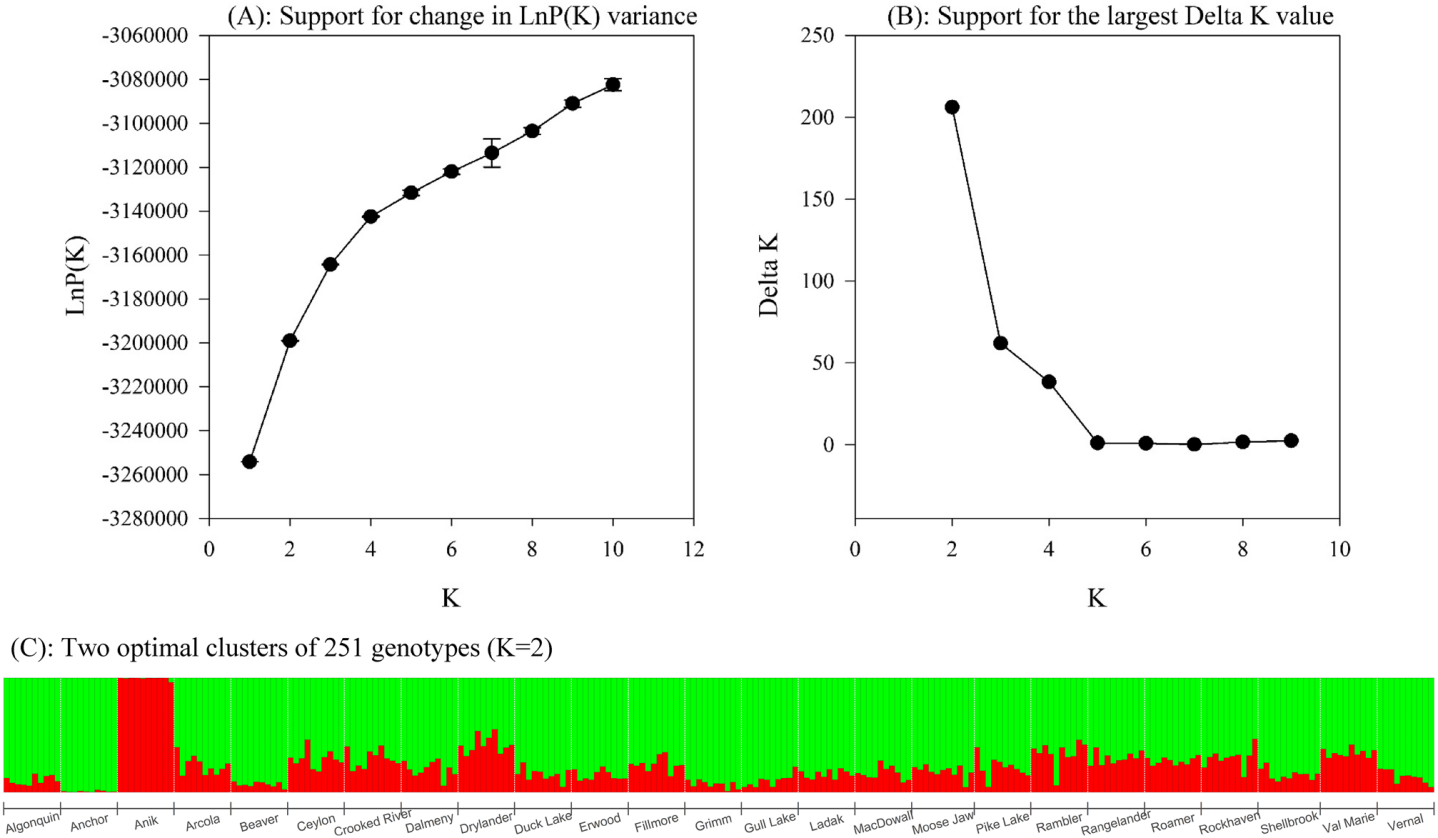
Genetic backgrounds inferred by STRUCTURE using 19,853 SNPs of 14 alfalfa populations from long-term grazing sites sampled across four soil zone of Saskatchewan Canada and 11 alfalfa cultivars released between 1926 and 1980. (**A**): Support for change in LnP(K) variance; (**B**): Support for the largest Delta K value; (**C**): Two optimal clusters of 251 genotypes (K = 2).

In STRUCTURE analysis, the optimum sub-population number was determined using the largest delta K value and the change in LnP(K) variance. Both methods confirmed that the optimal sub-population number was K = 2 (Fig. 1A and B), dividing the 25 populations (251 genotypes) into two main genetic backgrounds of *falcata* and *sativa* sub-groups (Fig. 1C). Based on the analysis, alfalfa cultivar Anik was a yellow flowering *falcata* sub-species. Anchor was a *sativa* sub-species with the highest *sativa* parentage. The genetic make-up of the majority populations and cultivars were intermediate to the two cultivars. Based on the individual genotype distribution, there was also two main groups. In Group I, there were 10 genotypes from Anik, and two from Drylander representing *falcata* genetic background (Fig. 1C). Group II was composed of 98 genotypes from 10 commercial alfalfa cultivars, and 141 genotypes from the 14 alfalfa populations from long-term grazing sites (Fig. 1C).

**Figure 2:**
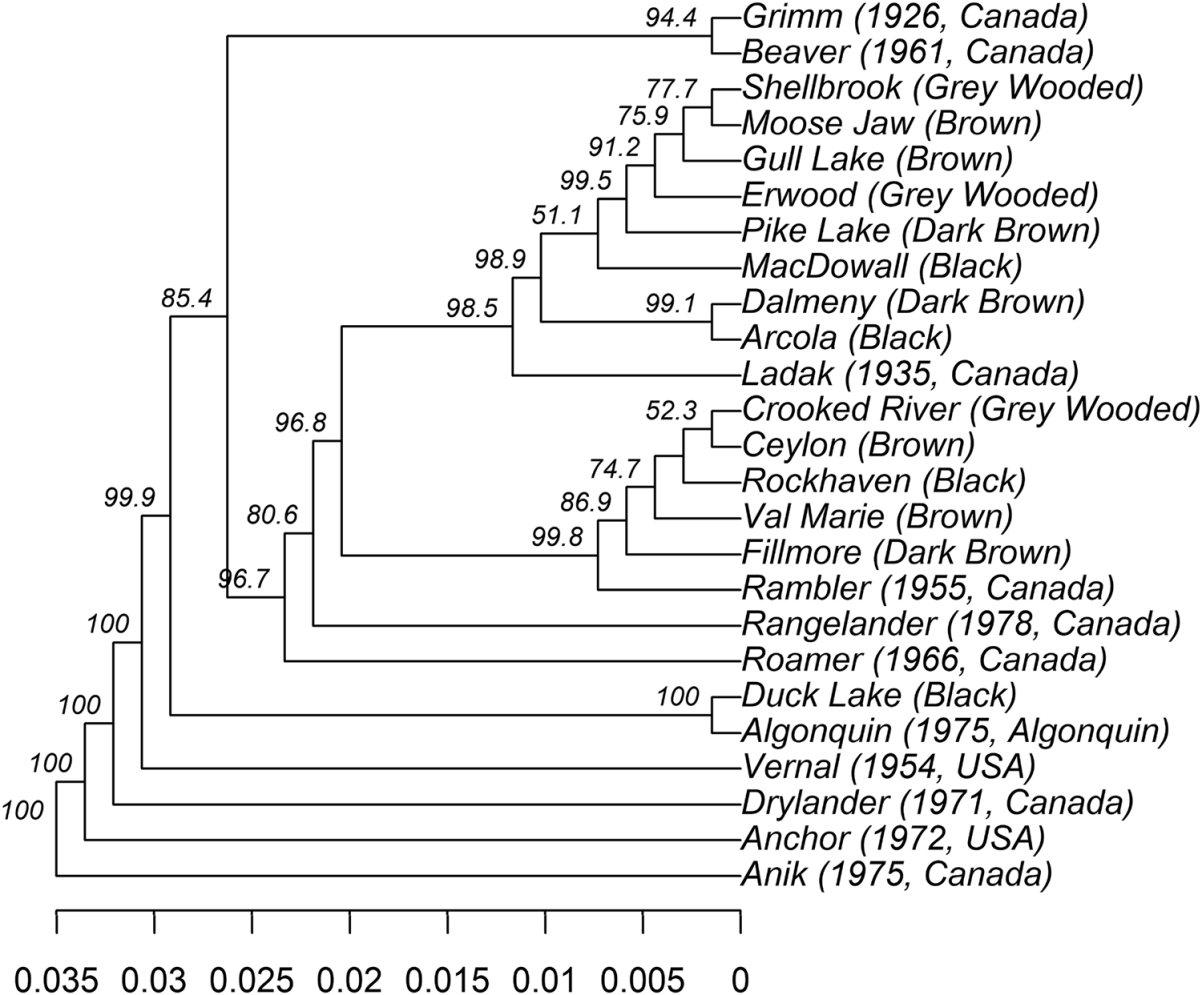
UPGMA tree based on Nei’s genetic distance among 25 alfalfa populations representing the 14 alfalfa populations from long-term grazing sites and 11 alfalfa cultivars released from 1926 to 1980; The original soil zone of 14 alfalfa populations from long-term grazing sites or the country and year of cultivars released are in the bracket. Bootstrap values (1000 replicates) are present for each node.

**Figure 3:**
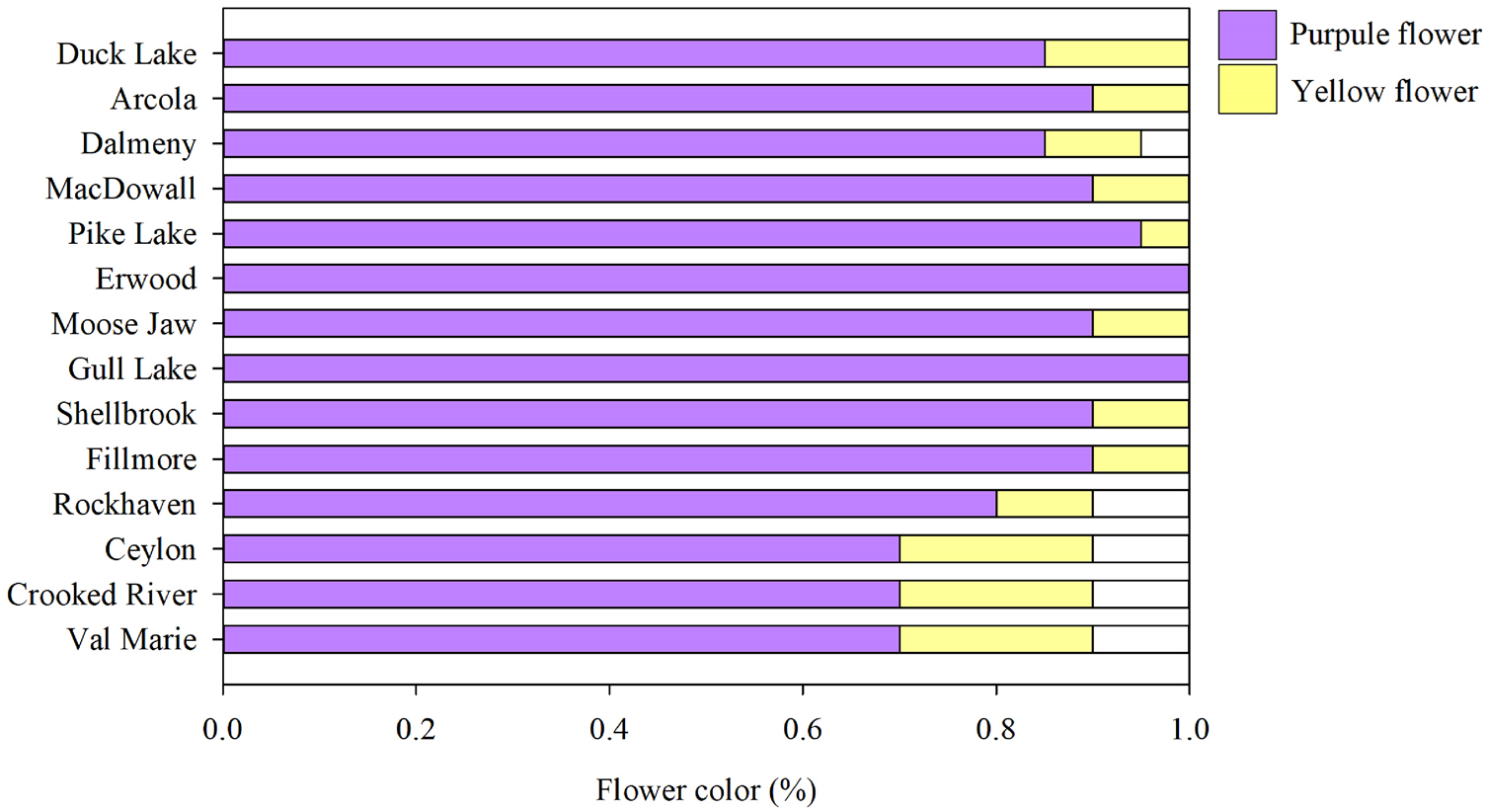
Mean flower color percentage in 14 alfalfa populations from long-term grazing sites.

**Figure 4:**
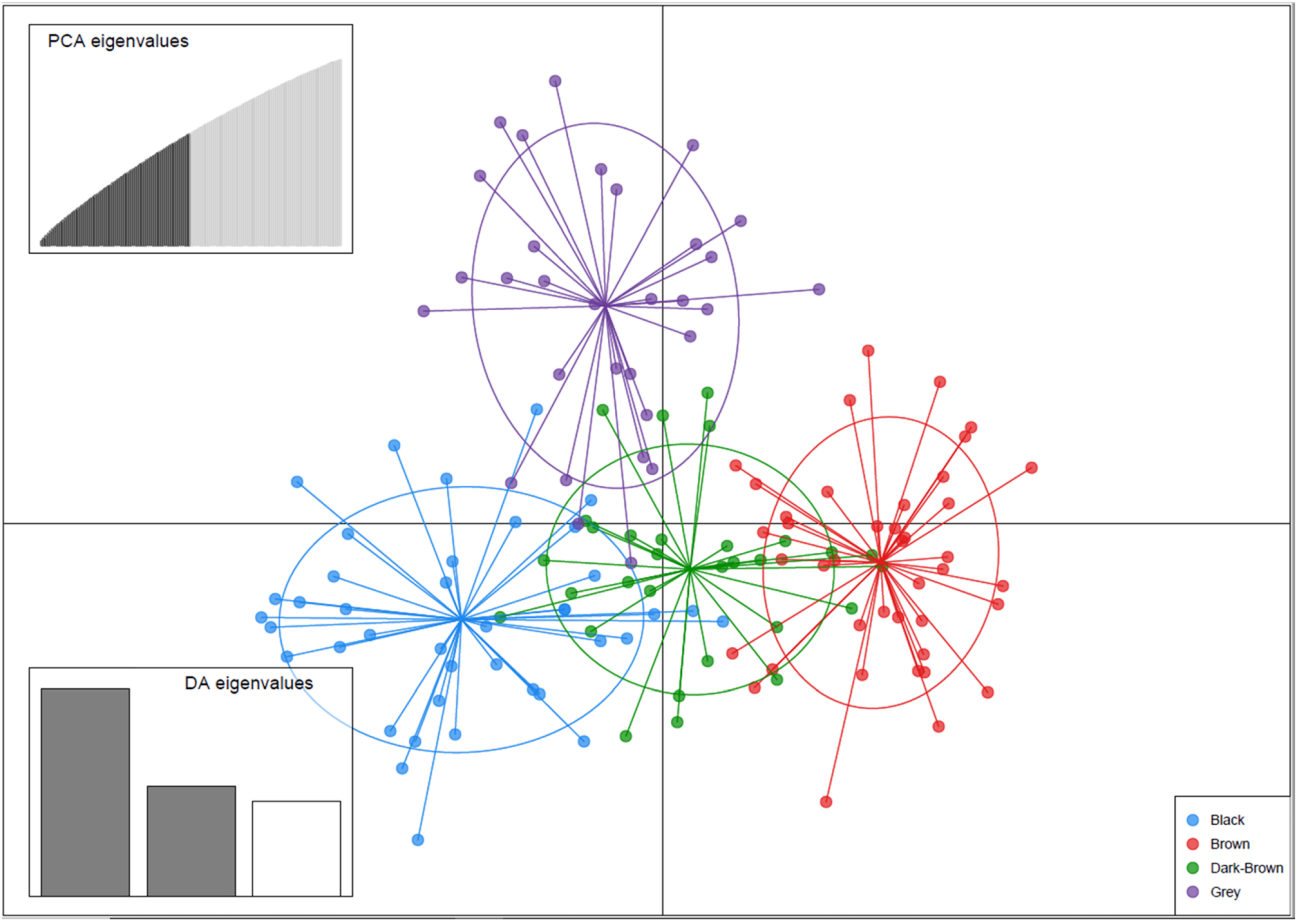
Discriminant analysis of principal component (DAPC) using the 19,853 SNPs of 141 alfalfa genotypes representing 14 alfalfa populations with long-term grazing history sampled across four soil zones of Saskatchewan, Canada. Each dot represents individual alfalfa genotype and the bottom right inset illustrates the legend for four soil zones.

Based on the UPGMA analysis, the populations from five grazed sites (Fillmore, Rockhaven, Ceylon, Crooked River, and Val Marie) were related to the old Canadian cultivar Rambler (Fig. 2). The Duck Lake population was closely related to the cultivar Algonquin, which is variegated light purple flowering type (Table 1S). Eight populations from various grazing sites (Shellbrook, Gull Lake, Moose Jaw, Erwood, Pike Lake, MacDowall, Dalmeny, and Arcola) were associated with the cultivar Ladak, which is a purple-flowered type (Table 1S). The UPGMA analysis was also supported by flower color-based clustering except Fillmore population (Fig. 3). The 14 alfalfa populations from long-term grazing sites were sampled from the four soil zones of Saskatchewan, and when we analyzed them by soil zone there was an apparent genetic divergence of populations by each soil zone, indicating a genetic shift by growth environment (Fig. 4).

### Phenotypic variation

There were significant differences for the six agro-morphological traits among the 14 alfalfa populations from long-term grazing sites (*p* < 0.001) and among the four soil zones (*p* < 0.01) (Tables 1 and 2). The MacDowall population had the highest forage dry matter yield, spring vigor, plant height, regrowth yield and fall plant height among them (Table 1). The Gull Lake population required the fewest growing degree days (GDDs) to produce the first flower, although it had 90% *sativa* parentage based on the STRUCTURE membership analysis. The most GDDs were needed for Ceylon population (29% *falcata* parentage) (Table 1 and Fig. 1C). Of the 14 populations, five of them (MacDowall, Dalmeny, Fillmore, Arcola and Rockhaven) were the top yielding populations (Table 1), while the top five rapid regrowing populations included Erwood, and Gull Lake in addition to the highest yielding populations (Table 1). The five early fall dormant populations based on fall plant height were Ceylon, Val Marie, Pike Lake, Rockhaven and Arcola, with *falcata* parentages of 29%, 34%, 21%, 30%, and 24%, respectively (Table 1 and Fig. 1C). The later dormant populations were MacDowall, Dalmeny, Duck Lake, Moose Jaw and Gull Lake, with *sativa* percentages of 84%, 80%, 85%, 82% and 90% (Table 1 and Fig. 1C).

**Table 1:** Mean comparison of six agro-morphological traits of 14 alfalfa populations with long-term grazing history among four soil zones from 2018–2020 grown at the Agriculture and Agri-Food Canada Saskatoon Research Farm, SK, Canada.

Population	Soil zone	Forage dry matter yield (g plant^−1^)	Spring vigor (1-5)	Days to flower (GDD)	Plant height (cm)	Regrowth (g plant^−1^)	Fall plant height (cm)
Crooked River	Grey Wooded	184^g^	2.2^f^	794^ab^	68^hi^	49^f^	12^ef^
Erwood	Grey Wooded	260^bc^	3.0^c^	703^f^	79^bcd^	81^b^	12^ef^
Shellbrook	Grey Wooded	246^cde^	2.9^cd^	731^ef^	76^def^	69^d^	12^ef^
Arcola	Black	260^bcd^	2.9^cd^	750^cde^	82^bc^	62^de^	11^f^
Duck Lake	Black	274^b^	3.4^b^	726^ef^	80^bc^	80^bc^	14^b^
MacDowall	Black	341^a^	3.6^a^	744^de^	90^a^	103^a^	17^a^
Rockhaven	Black	253^bcde^	3.0^c^	758^bcde^	76^ef^	63^de^	11^f^
Dalmeny	Dark Brown	253^bcde^	3.1^c^	778^bcd^	83^b^	70^cd^	15^b^
Fillmore	Dark Brown	232^ef^	2.9^cd^	784^abc^	74^fg^	64^d^	12^de^
Pike Lake	Dark Brown	234^de^	2.9^cd^	744^de^	79^cde^	55^ef^	12^ef^
Ceylon	Brown	205^fg^	2.1^f^	825^a^	70^gh^	50^f^	10^g^
Gull lake	Brown	185^g^	2.7^de^	730^ef^	71^gh^	62^de^	13^cd^
Moose Jaw	Brown	242^cde^	3.1^c^	757^bcde^	76^f^	66^d^	13^c^
Val Marie	Brown	189^g^	2.5^e^	792^ab^	65^i^	46^f^	12^ef^
SEM		14.3	0.3	14.3	2.1	6.0	0.4
*p* value		< 0.0001	< 0.0001	< 0.0001	< 0.0001	< 0.0001	< 0.0001

Note: The different lowercase letters within a column are significantly different according to Tukey’s HSD at *p* ≤ 0.05. SEM, standard error of the mean.

**Table 2:** Mean comparison of six agro-morphological traits of 14 alfalfa populations with long-term grazing history among four soil zones from 2018 to 2020 grown at the Agriculture and Agri-Food Canada Saskatoon Research Farm, SK, Canada.

Soil zone	Forage dry matter yield (g plant^−1^)	Spring vigor (1-5)	Days to flower (GDD)	Plant height (cm)	Regrowth (g plant^−1^)	Fall plant height (cm)
Black	282^a^	3.2^a^	745^b^	82^a^	77^a^	13^a^
Brown	205^c^	2.6^c^	776^a^	71^d^	56^c^	12^b^
Dark Brown	240^b^	3.0^b^	769^a^	78^b^	63^b^	13^a^
Grey Wooded	230^b^	2.7^c^	743^b^	75^c^	66^b^	12^b^
SEM	11.9	0.2	8.3	1.9	5.3	0.3
*p* value	< 0.0001	< 0.0001	< 0.01	< 0.0001	< 0.0001	< 0.0001

Note: The different lowercase letters within a column are significantly different according to Tukey’s HSD at *p* ≤ 0.05. SEM, standard error of the mean.

Among the four soil zones, the populations from the Black soil zone, the moistest region, had the highest forage yield, spring vigor, plant height, regrowth and fall plant height compared to the other three soil zones (Table 2). The populations from the Brown soil zone, the driest among four, had the lowest forage, spring vigor, plant height and regrowth (Table 2). The GDDs for days to flower was higher for the populations from two southern soil zones (Brown and Dark Brown soil zones) than the two northern zones (Black and Grey Wooded soil zones) (Table 2). Forage dry matter yield and regrowth were similar between populations from the Dark Brown and Grey Wooded soil zones (Table 2). The fall plant height was taller for the populations from the Black and Dark Brown soil zones than from the Brown and Grey Wooded soil zones (Table 2).

Significant variation existed among the 14 alfalfa populations from long-term grazing sites in each of the three nutritive value measurements (*p* < 0.01) and among the four soil zones (*p* < 0.001) (Tables 3 and 4). The concentrations of ADF ranged from 33.1 to 37.7%, and NDF ranged from 39.4 to 45.6%. The populations from Arcola and MacDowall had the highest concentrations of ADF and NDF, while populations from Crooked River had the lowest ADF and NDF concentrations (Table 3). The concentration of CP ranged from 16.2 to 18.6% among the 14 alfalfa populations from long-term grazing sites. The Crooked River population produced the highest CP concentration, followed by the Val Marie and Gull Lake populations (Table 3). Populations from the Black soil zone had the highest concentrations of ADF and NDF, while populations from the Brown and Grey Wooded soil zones had the lowest concentrations of ADF and NDF. The populations from Grey Wooded soil zones had the highest CP concentrations (Table 4).

**Table 3:** Mean comparison of three nutritive value traits of 14 alfalfa populations with long-term grazing history from 2018 to 2019 at the Agriculture and Agri-Food Canada Saskatoon Research Farm, SK, Canada.

Populations	ADF (%DM)	NDF (%DM)	CP (%DM)
Arcola	37.7^a^	44.8^a^	16.4^de^
Ceylon	34.9^cde^	41.6^def^	17.4^bc^
Crooked River	33.6^ef^	39.8^fg^	18.6^a^
Dalmeny	36^bc^	43.8^abc^	16.5^de^
Duck Lake	36.5^ab^	44.3^a^	16.2^e^
Erwood	35.6^bc^	41.9^cde^	17.3^bc^
Fillmore	34.7^cde^	40.8^efg^	17.6^b^
Gull lake	33.1^f^	40.0^fg^	17.7^b^
MacDowall	37.7^a^	45.6^a^	16.2^e^
Moose Jaw	34.2^def^	41.3^defg^	16.9^cd^
Pike Lake	35.7^bc^	42.6^bcd^	16.4^de^
Rockhaven	36.6^ab^	43.9^ab^	16.5^de^
Shellbrook	35^cd^	42.6^bcd^	16.7^de^
Val Marie	33.2^f^	39.4^g^	17.9^b^
SEM	0.7	0.6	0.2
*p* value	< 0.01	< 0.01	< 0.0001

Note: The different lowercase letters within a column are significantly different according to Tukey’s HSD at *p* ≤ 0.05. SEM, standard error of the mean.

**Table 4:** Mean comparison of three nutritive value traits among four soil zones for 14 alfalfa populations with long-term grazing history from 2018 to 2019 at the Agriculture and Agri-Food Canada Saskatoon Research Farm, SK, Canada.

Soil zone	ADF (%DM)	NDF (%DM)	CP (%DM)
Black	37.1^a^	44.6^a^	16.3^c^
Brown	33.9^c^	40.6^c^	17.5^a^
Dark Brown	35.5^b^	42.4^b^	16.9^b^
Grey Wooded	34.7^b^	41.4^bc^	17.5^a^
SEM	0.6	0.3	0.1
*p* value	< 0.0001	< 0.0001	< 0.0001

Note: ADF, acid detergent fiber; NDF, neutral detergent fiber; CP, crude protein. The different lowercase letters within a column are significantly different according to Tukey’s HSD at *p* ≤ 0.05. SEM, standard error of the mean.

**Figure 5:**
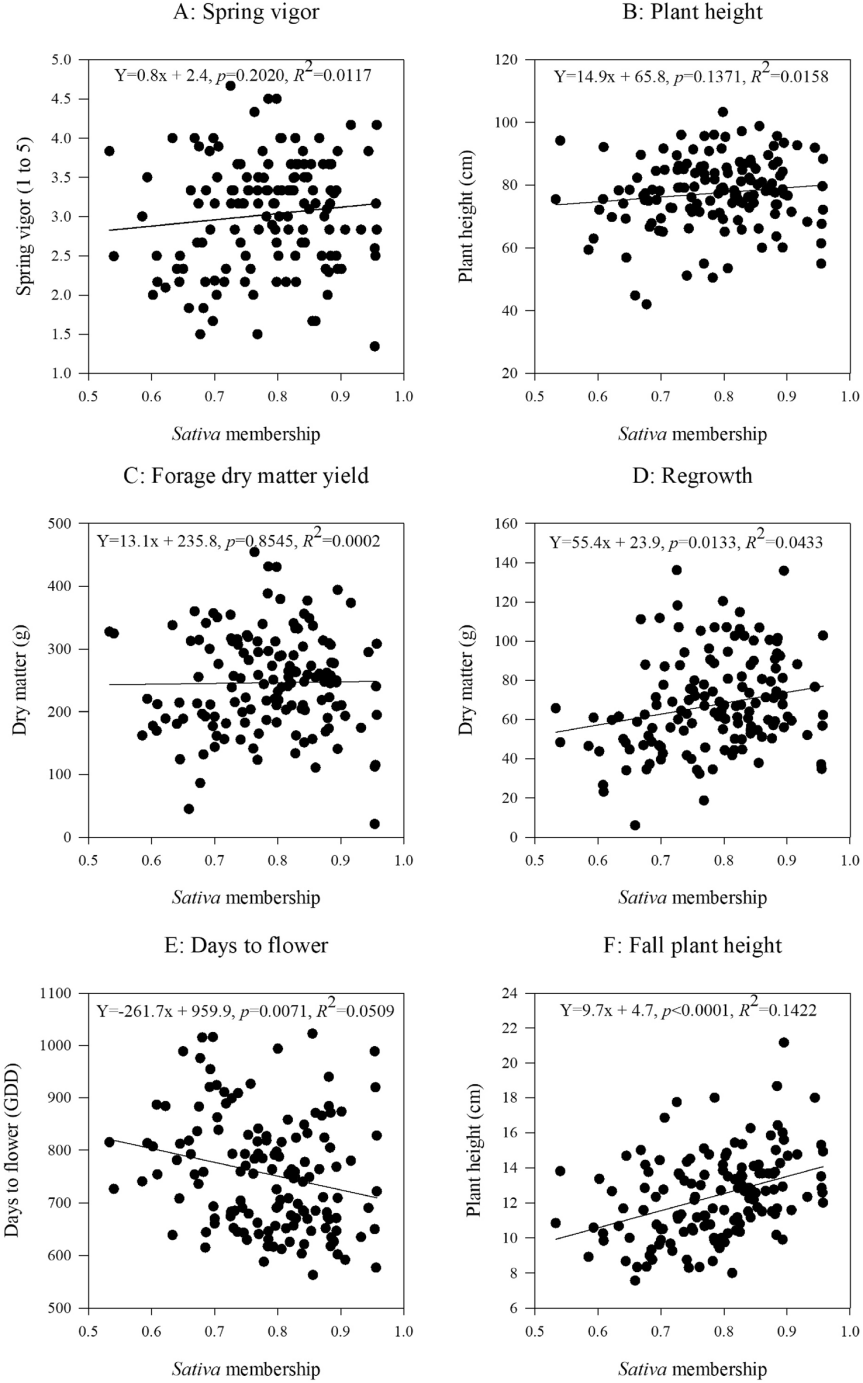
Relationship between STRUCTURE based *sativa* parentage (%)with six morphological traits of 14 alfalfa populations from long-term grazing sites.

**Figure 6:**
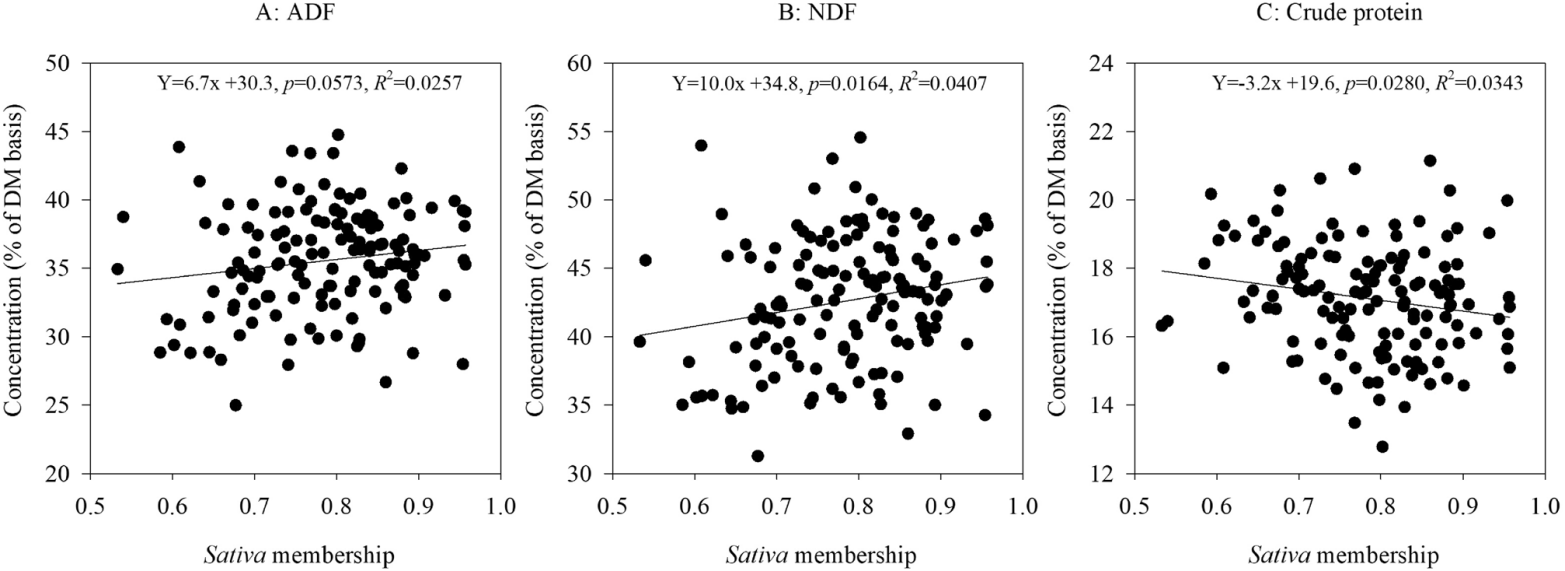
Regression between STRUCTURE *sativa* parentage (%) and values of three nutritive traits of 14 alfalfa populations from long-term grazing sites.

**Figure 7:**
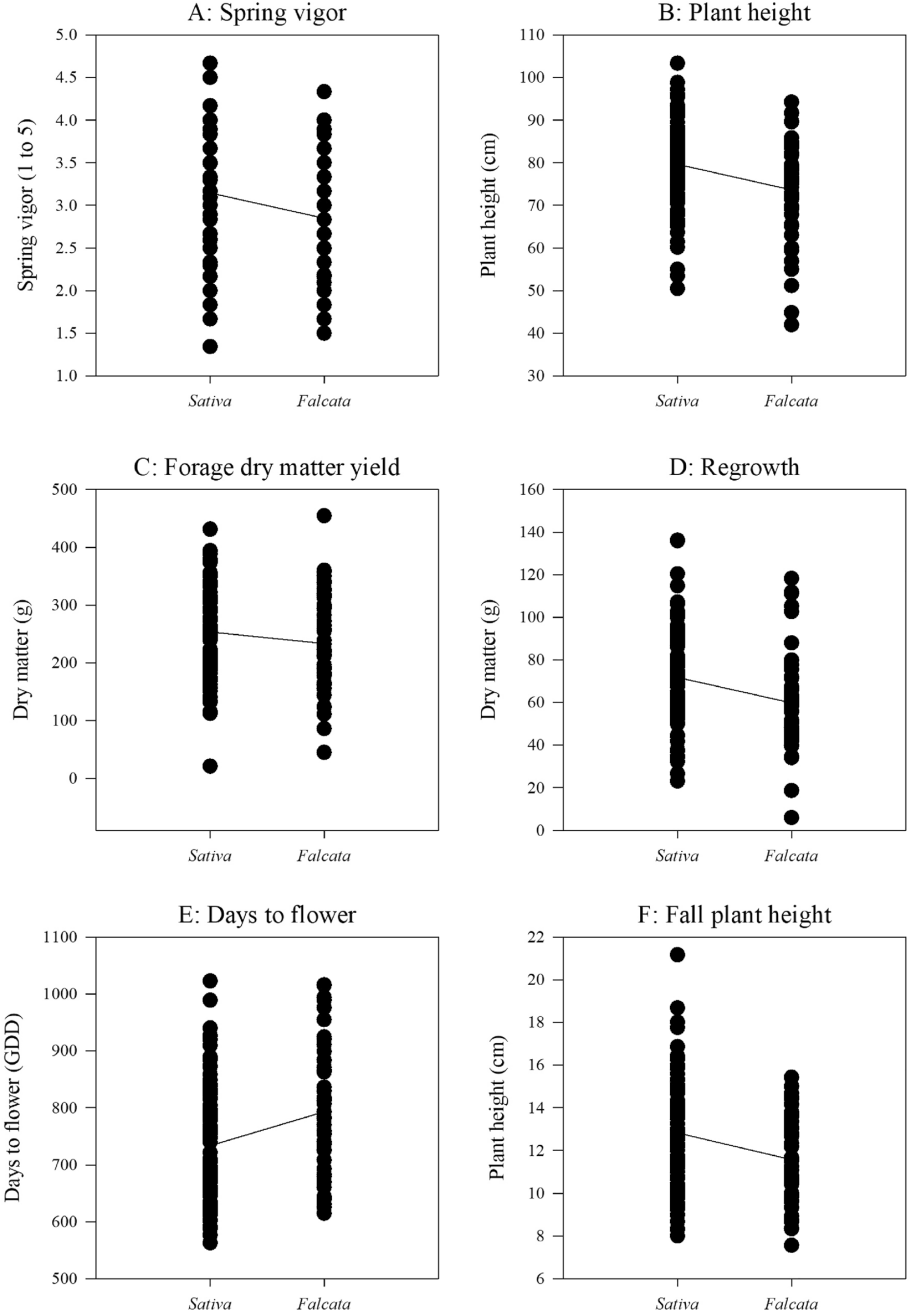
Trend of six morphological traits changes between *sativa* and *falcata* sub-clusters of 14 alfalfa populations with long-term grazing history.

**Figure 8:**
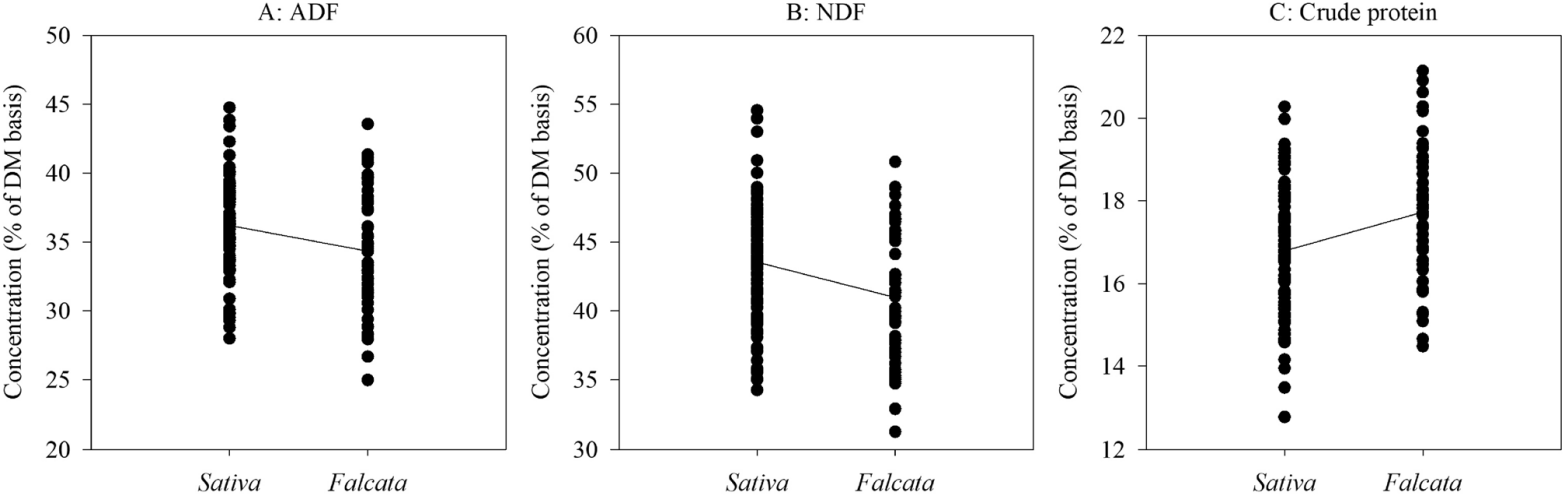
Trend of three nutritive value traits changes between *sativa* and *falcata* sub-clusters of 14 alfalfa populations with long-term grazing history.

We grouped genotypes of the 14 alfalfa populations from long-term grazing sites into *sativa* and *falcata* sub-groups based on the percent of *sativa* (or *falcata*) parentage in STRUCTURE analysis. If a genotype is made up of greater than 50% *sativa* parentage, and then it will be grouped into *sativa* cluster, and vice versa (Fig. 1C). The linear regressions of STRUCTURE based *sativa* parentage percent (membership probability) on phenotypic values of forage dry matter yield, spring vigor and plant height were not significant (Fig. 5), while the phenotypic values of fall plant height, regrowth, days to flower, NDF and CP were significantly associated with their STRUCTURE *sativa* membership probability (Figs. 5 and 6). Based on the UPGMA results, the *sativa* populations (Duck Lake, Shellbrook, Gull Lake, Moose Jaw, Erwood, Pike Lake, MacDowall, Dalmeny, and Arcola) had higher phenotypic values of spring vigor, plant height, forage dry matter yield, fall plant height, ADF and NDF but lower values of days to flower and CP as compared to the *falcata* populations (Fillmore, Rockhaven, Ceylon, Crooked River and Val Marie) (Figs. 7 and 8).

### Genotype-environment association (GEA)

#### GEAs for soil nutrients

A total of seven significant GEAs were identified for soil K on chromosomes 1.3, 1.4, 2.4, 4.2, and 8.1 (Table 5). The correlations between soil K with allele frequencies at these SNPs ranged from 0.17 to 0.37. Of these, one SNP on chromosome 2.4 located at 28.5 Mb had the highest correlation of 0.37 and was related to MS.gene77592 encoding Cytochrome P450. Four significant GEAs were identified for soil P on chromosomes 1.4, 3.2 and 7.4 (Table 5). The correlations at these SNPs ranged between 0.24 and 0.33. The SNP on chromosome 3.2 located at 49.3 Mb had the highest correlation of 0.33 and was associated with MS.gene064294 encoding ABC transporter type 1, transmembrane domain superfamily. The SNP on chromosome 1.4 was related to MS.gene46469 encoding Leucine-rich repeat, cysteine-containing subtype. A total of four significant GEAs were identified for soil S on chromosomes 3.2 and 4.3 (Table 5). The correlations at these SNPs ranged from 0.22 to 0.24. One SNP on chromosome 3.2 at 42.9 Mb indicated the highest correlation of 0.24 and was related to MS.gene30119 encoding Armadillo-like helical. The three other SNPs on chromosome 4.3 located at 7.1 Mb had correlation of 0.22 and were related to MS.gene000431 encoding Ankyrin repeat-containing domain. Four significant GEAs were identified for soil pH on chromosomes 3.1, 4.3 and 7.4 (Table 5). The correlations at these SNPs ranged from 0.24 to 0.34 with a SNP on chromosome 3.1 at 74.9 Mb showing the highest correlation of 0.34. This SNP was associated with MS.gene049423 encoding Zinc finger, RING-type. In addition, the correlations at two other SNPs on chromosome 4.3 located at 7.1 Mb were 0.24. The SNP on chromosome 4.3 located at 7.1 Mb had correlation of 0.24 and was related to the same gene MS.gene000431 for soil pH (Table 5). Another SNP on chromosome 4.3 located at 7.1 Mb was related to MS.gene000426 encoding WD40 repeat (Table 5).

**Table 5:** Candidate genes associated with six environmental factors of long-term grazing sites detected by redundancy analysis (RDA).

Environmental predictor	Chromosome	SNP position (bp)	− log_10_(*p*-value)	Correlation	Gene	Annotation
K	1.3	7830256	4.70	0.23	MS.gene051501	TMEM85/ER membrane protein complex subunit 4
	1.3	7830268	4.70	0.23	MS.gene051501	TMEM85/ER membrane protein complex subunit 4
	1.4	15642882	5.68	0.26	Intergenic region	
	2.4	28599385	4.64	0.37	MS.gene77592	Cytochrome P450
	4.2	70902503	4.88	0.31	MS.gene043198	S-phase kinase-associated protein 1
	4.2	70902518	4.89	0.25	MS.gene043198	S-phase kinase-associated protein 1
	8.1	61223859	6.05	0.17	MS.gene063930	Protein of unknown function
P	1.4	45061295	6.05	0.25	MS.gene46469	Leucine-rich repeat, cysteine-containing subtype
	3.2	49315737	4.74	0.33	MS.gene064294	ABC transporter type 1, transmembrane domain superfamily
	7.4	37418579	5.30	0.24	MS.gene055024	Unknown function
	7.4	37418633	6.03	0.25	MS.gene055024	Unknown function
S	3.2	42941661	5.12	0.24	MS.gene30119	Armadillo-like helical
	4.3	7114035	6.35	0.22	MS.gene000431	Ankyrin repeat-containing domain
	4.3	7114063	6.35	0.22	MS.gene000431	Ankyrin repeat-containing domain
	4.3	7114088	6.35	0.22	MS.gene000431	Ankyrin repeat-containing domain
Soil pH	3.1	74939320	5.36	0.34	MS.gene049423	Zinc finger, RING-type
	4.3	7164352	5.34	0.24	MS.gene000431	Ankyrin repeat-containing domain
	4.3	7164366	5.34	0.24	MS.gene000426	WD40 repeat
	7.4	49716258	5.13	0.32	Intergenic region	
Summer extreme temperature	1.1	15400489	4.63	0.26	Intergenic region	
	1.1	15400560	4.63	0.26	Intergenic region	
	4.4	10075347	7.85	0.38	MS.gene28113	FKBP-type peptidyl-prolyl cis–trans isomerase domain
	4.4	10075356	7.85	0.38	MS.gene28113	FKBP-type peptidyl-prolyl cis–trans isomerase domain
	4.4	8735841	8.80	0.26	MS.gene28435	MORN motif
	5.2	53873180	5.64	0.21	Intergenic region	
	5.2	53916206	5.56	0.21	Intergenic region	
	8.1	61223799	5.78	0.14	MS.gene063930	Unknown function
Winter extreme temperature	4.3	7164382	5.70	0.23	MS.gene000426	WD40 repeat
	7.1	62118779	5.49	0.35	MS.gene30010	XPG/Rad2 endonuclease
	8.1	9987197	4.99	0.27	MS.gene58730	Alpha/Beta hydrolase fold
	8.1	9987210	4.99	0.27	MS.gene58730	Alpha/Beta hydrolase fold

#### GEAs for summer and winter extreme temperature

A total of eight significant GEAs were identified for summer extreme temperature on chromosomes 1.1, 4.4, 5.2 and 8.1 (Table 5). The correlations at these SNPs with summer extreme temperature ranged from 0.14 to 0.38. Two SNPs on chromosome 4.4 at 10.0 Mb had the highest correlation of 0.38 and were related to same gene MS.gene28113 encoding FKBP-type peptidyl-prolyl cis–trans isomerase domain. The SNP on chromosome 4.4 at 8.7 Mb had correlation of 0.26 and was related to MS.gene28435 encoding MORN motif. Moreover, four significant GEAs were identified for winter extreme temperature on chromosomes 4.3, 7.1, and 8.1 (Table 5). The correlations at these SNPs ranged from 0.23 to 0.35. One major SNP on chromosome 7.1 at 62.1 Mb had the correlation of 0.35 and was related to MS.gene30010 encoding XPG/Rad2 endonuclease. The SNP on chromosome 4.3 at 7.1 Mb had a correlation of 0.23 and was related to the same gene MS.gene000426 for soil pH (Table 5). Two SNPs on chromosome 8.1 at 9.9 Mb had the same correlation of 0.27 and were related to MS.gene58730 encoding Alpha/Beta hydrolase fold (Table 5).

To estimate r^2^ values of SNPs within the same gene identified in GEA results, LD was analyzed using a total of 12,402 SNP markers. The r^2^ of LD across all chromosomes was calculated and is presented in Fig. 1S (see the Supplementary files). A rapid drop in r^2^ was observed with the increase in physical distance between 0 and 5 cM, and then r^2^ showed no decrease afterward (Fig. 1S). Meanwhile, five pairs of SNPs were found within same gene: two for soil K, one for soil S, one for winter extreme temperature, and one for summer extreme temperature (Fig. 2S). The physical distances for these SNP pairs ranged from 12 to 54 bp, and the r^2^ values were greater than 0.9 (Fig. 2S).

## Discussion

### Population structure

The STRUCTURE analysis confirmed two optimal clusters among 251 alfalfa genotypes representing the 14 alfalfa populations from long-term grazing sites and the 11 commercial alfalfa cultivars widely seeded from 1926 to 1980 in western Canada. The Cluster I included genotypes with a high percentage of *falcata* background, supported by cultivar Anik (known as *falcata* population) as an independent cluster with two genotypes from cultivar Drylander. *M. falcata* has yellow flowers and uncoiled seed pods, and is widely considered to be indigenous to Siberia^28^. The subspecies has also shown slow regrowth and creeping root-habit^29,30^, with good drought, extreme winter hardiness and grazing resistance^3,31^. The cultivars Rambler, Roamer and Rangelander have intermediate levels of *falcata* backgrounds, which is consistent with their pedigrees. For example, Rambler was recorded as having 45% of *M. falcata* parentage^31^. *M. falcata* was used as one of parents for artificial crossing with the cultivar Ladak (a purple flowered *M. sativa*) to develop creeping-rooted cultivars for grazing in western Canada, which resulted in Rambler^32^, Roamer^33^ and Drylander^34^. Rangelander was selected from a cross among Rambler, Roamer, Drylander and strains of *M. falcata*^35^. The Cluster II was composed of genotypes with high percentages of *M. sativa* background including cultivars of Algonquin, Anchor, Beaver, Grimm, Ladak, and Vernal. *M. sativa* is a purple-flowered form with more rapid growth, and a relatively more erect growth form than *M. falcata*^3,22,36,37^. The cultivar Anchor is considered as typical of the *M. sativa* subspecies, while Beaver alfalfa has 98% of *M. sativa* parentage^31^. Algonquin showed 50% of *M. sativa* parentage, since this cultivar was developed by open pollinating *M. media* (*M. falcata* × *M. sativa*) to the cultivar Rhizoma^38^, which had 50% parentage from *M. falcata*, and another 50% from Grimm (*M. media*)^31^.

Interestingly, both DAPC and genetic distance analyses showed no distinctive separation of the 14 alfalfa populations from long-term grazing sites. Initially, we expected that the 14 populations would be associated with one of the 11 cultivars used in the region at that time. This result might be caused by a genetic shift in response to regional environments^39^, and long-term grazing managements^8–11,39^. The 14 alfalfa populations from long-term grazing sites had at least 25 years of utilization by harvesting hay or grazing annually, which affected their population genetic diversity. However, the DAPC analysis on variability by soil zone showed a genetic shift of the 14 populations by soil zone. This might be attributed to interactive effects of climatic factors (precipitation and temperature), differential grazing pressure and soil properties such as texture, pH, and nutrient levels on genetic variation of the alfalfa populations. Blanco-Pastor et al.^16^ found a set of candidate genes associated with local environmental factors for diploid alfalfa populations, confirming local adaptation. In addition, long-term grazing activities may have also changed genetic structure. Although there are no reports of genetic shift of alfalfa populations under grazing, a few studies indicated that intensive grazing decreased genetic diversity in forage grasses, i.e., *Stipa grandis*^8^, *Elymus nutans* Griseb.^9^, blue grama (*Bouteloua gracilis*)^10^, and meadow fescue (*Festuca pratensis* Huds.)^11^.

When analyzed by populations based on the UPGMA analysis, five alfalfa populations from the 14 long-term grazing sites (Val Marie, Crooked River, Ceylon, Rockhaven and Fillmore) were genetically related to the cultivar Rambler. This cultivar is a variegated flower type with a higher level of parentage of *M. falcata* than Roamer and Rangelander^32^. This cultivar was superior in drought resistance and winter hardiness to Ladak and Grimm, but has slow recovery after cutting or grazing than either variety^32^. It was selected for improved drought tolerance, the climatic conditions often found in the Brown soil zone^40^. Thus, it would not be surprising to find this cultivar at the Ceylon, Val Marie sites, which are located in the Brown soil zone. The population from Duck Lake was genetically associated with the cultivar Algonquin. This cultivar is a variegated light purple type with high of parentage of *M. sativa*. Algonquin was developed by backcrossing plants resistant to bacterial wilt (*Corynebacterium insidiosum* (McCull) H. L. Jens) into the cultivar Rhizoma^38^. Eight alfalfa populations from the long-term grazing sites were genetically related to Ladak, which is a purple-flowered type which has a greater percent of *M. sativa* parentage. In the history of alfalfa cultivation under northern Great Plains conditions, Ladak not only outyielded Grimm but also had a longer stand persistence than other alfalfa cultivars at that time due to its excellent cold and drought tolerances^41^. *M. sativa* generally has higher forage yield, faster regrowth, and a relatively more erect growth form than *M. falcata*^3,22,36,37^. These populations were generally from the Black or Dark Brown soil zones where soil moisture is higher than the Brown soil zone.

### Phenotypic variation

Significant differences were observed for six agro-morphological and three nutritive values traits among the 14 alfalfa populations with long-term grazing history. These traits were related to their relative levels of parentage to either *falcata* or *sativa* sub-species. For example, based on the genetic background inferred by UPGMA analysis, the mean forage dry matter yield, regrowth and yield-related traits (e.g., plant height, fall plant height and spring vigor), ADF and NDF of alfalfa populations with long-term grazing history in the sub cluster with Ladak and Algonquin were higher than those in the subcluster with Rambler, while days to flower and CP were lower. The subcluster with Ladak and Algonquin with a high percentage of *M*. *sativa* genome, while the subcluster with Rambler had a high percentage of *falcata* genome. The *M. sativa* genome has been considered to provide the genetic basis for high forage dry matter yield, rapid regrowth and low fall dormancy as compared with the *M. falcata* genome^7,30,42,43^. Interestingly, the alfalfa populations with a greater parentage from the *M. falcata* genome (Crooked River, Val Marie, Rockhaven, Fillmore and Ceylon) required longer GDDs and produced higher concentrations of CP and lower concentrations of ADF and NDF. The alfalfa cultivar AC Yellowhead, belonging to the *falcata* sub-species, has been shown to contain higher CP concentration to Beaver alfalfa cultivar, a *M. sativa*^44^ cultivar. Meanwhile, significant differences were also observed for the agro-morphological traits and nutritive values among the four soil zones. The alfalfa populations from the Black soil zone produced the highest forage dry matter yield, spring vigor, plant height, regrowth and fall plant height, while the populations from the Brown soil zone had the lowest values for agro-morphological traits. This is because that three of four alfalfa populations (Duck Lake, Arcola and MacDowall) in the Black soil zone were identified as having high percentages of the *M. sativa* genome based on the UPGMA analysis. By contrast, two of four alfalfa populations (Ceylon and Val Marie) in the Brown soil zone were identified as having high percentages of *M. falcata* genome.

The GDDs required for developing flowers for the 14 alfalfa populations from long-term grazing sites increased gradually in the order of Grey Wooded < Black < Dark Brown < Brown soil zones. This is an interesting regional adaptation trait as a reduced number of GDDs requirement for the populations from the Grey-Wooded and Black soil zones has an ecological significance for maintaining plant population and its genetic diversity by sexual reproduction. These regions have colder springs, and are at higher latitudes (longer days) than the Brown soil zone sites. Day-length and temperature are the two main environmental factors influencing flower development^45^. Alfalfa is a perennial, long-day plant, and its flowering is induced when the photoperiod exceeds a critical threshold value^46^. Lower accumulated temperature has been shown to delay flowering time for alfalfa^47^. Knowledge on environmental and genetic factors regulating flowering time can help farmers to balance forage quality and yield^48^.

The linear regression of STRUCTURE membership probability on phenotypic values indicated that there was lower genetic variation of first cut forage yield (hay yield) and yield-related traits (spring vigor and plant height) as *sativa* parentage percent increased. This result may be explained by recurrent phenotypic selection on forage dry matter yield and related traits that has been carried out in the past. However, the phenotypic variation of the traits regrowth, fall plant height, days to flower, NDF and CP were significantly associated with higher *sativa* percentage, which indicated that high genetic variation of these five traits existed in the 14 alfalfa populations with long-term grazing history. These findings may suggest that less phenotypic selection was carried out on these five traits in past breeding programs.

### Genotype-environment association

The GEA analysis identified 31 SNPs highly associated with six environmental factors in the alfalfa populations from long-term grazing sites. Of the 31 SNPs identified, eight SNPs were associated with the summer extreme temperature, which is a critical factor affecting alfalfa production in western Canada^49^. In most cases, the summer extreme temperatures have been accompanied by drought stress in western Canada^22,50–52^. Historically, drought stress has occurred frequently in the Brown soil zone^53^, and drought could become more common under climate change^54^. Therefore, these genes may be useful for marker assisted breeding of climate resilient cultivars. Two major SNPs identified on chromosome 4.4 were linked with the putative candidate gene, MS.gene28113 (*FKBP-type peptidyl-prolyl cis–trans isomerase domain*) and MS.gene28435 (*Membrane occupation and recognition nexus motif*). It has been reported that the peptidyl prolyl cis–trans-Isomerase *FKBP77* gene is involved in heat stress tolerance^55^. The PI monophosphate 5-kinase (PIP5K) enzyme containing membrane occupation and recognition nexus motif is involved in heat and drought-responses in plants^56,57^.

In western Canada, alfalfa dry matter yield is often limited by winter injury^58^, which can be caused by fall cutting or grazing, resulting low root organic matter^20^. *M. falcata* with strong winter hardiness has been used to breed alfalfa for improved winter survival^31^. Among the four SNPs associated with winter extreme temperature, the SNP on chromosome 4.3 was the most significant, which was linked to the putative candidate gene MS.gene000426 (*WD40 repeat*). The WD40 gene *OsTTG1* regulates anthocyanin biosynthesis, which plays an important role in the growth of plants and is affected by temperature^59–63^.

Potassium (K) promotes alfalfa persistence and stand longevity^64–67^. Alfalfa plants deficient with K had low root organic reserves for new growth and maintenance^67–69^. Of seven significant SNPs, one major SNP on chromosome 2.4 is linked to the candidate gene MS.gene77592 (*Cytochrome P450*), which is involved in glucosinolate synthesis^70–72^. Glucosinolate plays an important role in plant response to abiotic stress^73^. Another two SNPs on chromosome 4.2. The putative candidate gene linked with these two markers is MS.gene043198 (*S-phase kinase-associated protein 1*). Plant SKP1-like family proteins have been found to associate with the regulation of plant alkaline tolerance and ABA sensitivity^74^. These two candidate genes identified here had no direct relationship with K stress in plants. This may be because the soils in our study were not K deficient based on soil tests.

Sulfur (S) has been reported to decrease the survival of alfalfa seedlings^75^. Four significant SNPs were identified for S on chromosome 3.2 and 4.3. Several repeat protein gene families have been identified in plants. The SNP on chromosome 3.2 is linked to the putative candidate gene MS.gene30119 (*Armadillo-like helical*). Some studies have reviewed the importance of the relationship between S assimilation and phytohormones^76,77^. Phytohormones are essential for plant acclimation and adaptation to environmental changes^78^. The other three SNPs on chromosome 4.3 were linked with MS.gene000431 (*Ankyrin repeat-containing domain*). The Ankyrin repeat domain C3HC4-Type RING finger genes play important roles in various physiological processes of plants^79^.

Phosphorus (P) is a vital macronutrient that plays important roles in energy transfer, respiration, enzyme activation, photosynthesis, and root elongation^80^. Low soil P availability is a major limiting factor in old alfalfa stands, which was verified by our soil nutrient analyses at these long-term sites. Four significant SNPs were identified for P in our study with a major SNP on chromosome 3.2 located at 49.3 Mb. The putative candidate gene linked with this marker is MS.gene064294 (*ABC transporter type 1, transmembrane domain superfamily*). ATPase binding cassette (ABC) transport G subfamily plays a crucial role in the transportation of biological molecules across the membrane. Overexpression of *L.albABCG29* in white lupin hairy root enhanced P accumulation in cluster roots under low P and improved plant growth^81^. Another SNP on chromosome 1.4 located at 45.0 Mb had a correlation of 0.25, and the putative candidate gene linked with this marker is MS.gene46469 (*Leucine-rich repeat, cysteine-containing subtype*).

Alfalfa plants are sensitive to acid soil^82^. The collected sites of the 14 alfalfa populations from long-term grazing sites had soil pH > 7. Four significant SNPs were identified for soil pH with a major SNP on chromosome 3.1. The putative candidate gene linked with this marker is MS.gene049423 (*Zinc finger, RING-type*). NCA1 gene encoding RING-type zinc finger domain regulates other protein activities in response to alkaline stress^83^. Two different SNPs on chromosome 4.3 were linked to the putative candidate genes MS.gene000431 (*Ankyrin repeat-containing domain*) and MS.gene000426 (*WD40 repeat*), respectively. The WD40 related proteins are involved in plant resistance to alkali stress^84^. The genes encoding ankyrin repeat domain has been reported to be associated with salinity-alkali stresses^85^. Our results showed that the LD distance within the alfalfa populations with long-term grazing history was larger (r^2^ reduced to 0.2 after 3 cM) than previous studies^24,86,87^. One main reason is that the alfalfa populations with long-term grazing history in our study were from genetically related alfalfa cultivars, and then low chromosome recombination events occurred within these cultivars.

This study identified 13 candidate genes associated with six environmental factors, reflecting adaptation of the 14 alfalfa populations from long-term grazing sites to their given environments across different soil zones. Most of these genes play an important role in plant tolerances to biotic and abiotic stresses, such as heat, drought, cold, salt and diseases. In western Canada, extreme low temperature^17–21^, and frequent drought stresses^5,22,37,50–52,88^ can significantly reduce alfalfa forage yield and stand persistence, acting as a natural selection for local adaptation. Inevitably, the majority of original alfalfa plants at the 14 long-term grazing sites might have died because of lack of adaption to interaction of grazing and environmental conditions (i.e., extreme drought in summer, extreme low temperature in winter or diseases). Similar to the results in our study, a number of studies reported candidate loci responsible for adaptation to climate gradients in diploid alfalfa^16^, *M. truncatula*^16,39,89,90^ and perennial ryegrass^91^. In addition, Humphries et al.^92^ demonstrated that collecting alfalfa germplasms that have evolved to survive in extreme environments with low rainfall, high temperature, and winter freezing would be one option to breed alfalfa adapted to the warming climates around the world. Therefore, the collected alfalfa populations with higher forage dry matter in our study can be a valuable genetic resource for future alfalfa breeding. Meanwhile, the 13 candidate genes involved in regulation of alfalfa plants’ response to environmental factors need to be validated in the future experiments, and then these genes can be used for marker-assisted selection for alfalfa breeding and genetic improvement.

## Methods

### Plant materials and soil sampling

**Figure 9:**
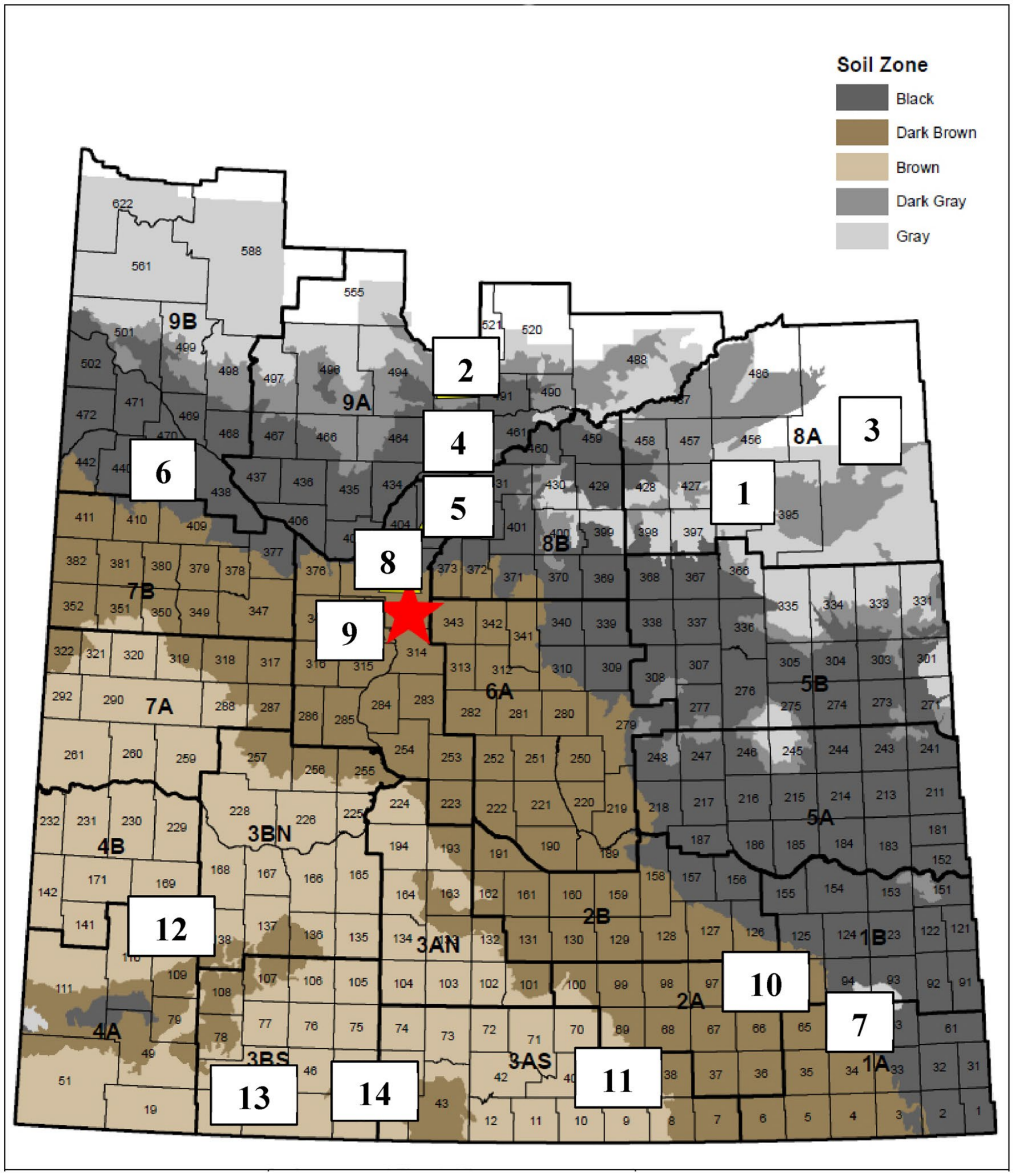
Locations of 14 long-term grazing sites for alfalfa plant selection during this study. (Sites names 1–14 are: Crooked River, Shellbrook, and Erwood; MacDowall, Duck Lake, Rockhaven and Arcola; Dalmeny, Pike Lake, and Fillmore; Ceylon, Gull Lake, Moose Jaw, and Val Marie). Map was adapted from Canadian Soil Information Service (https://sis.agr.gc.ca/cansis/index.html).

In spring 2016, 14 alfalfa-grass mixed stands with a minimum of 25 years of grazing history were selected across Brown, Dark Brown, Black and Grey Wooded soil zones of Saskatchewan, Canada (Table 6; Fig. 9). Our research team obtained permissions to collect cultivated alfalfa plants from the 14 private ranchers prior to this study. The plant materials are not wild plants or endangered species. Experimental research and field studies on plants including the collection of plant material, complied with relevant institutional, national, and international guidelines and legislation. The Brown soil zone is the most arid region characterized by annual precipitation in the range of 275–325 mm. It has the highest evapotranspiration among the four soil zones. The Dark Brown soil zone is characterized by annual precipitation in the range of 325–375 mm. It has a moderate evapotranspiration. The Black soil zone is characterized by annual precipitation in the range of 400–475 mm. The Grey Wooded soil zone is characterized by annual precipitation ranging from 300 to 500 mm with lower evapotranspiration than in the Black soil zone^40^. At all grazing sites, alfalfa plants were grown in a grass-alfalfa mixture, which were grazed at least once a year over 25 years. At each site, 30 mature alfalfa plants were dug and stored in a plastic container with 10 cm water before transplanting to a spaced nursery at Saskatoon, Saskatchewan for agronomic traits evaluation. The distance between any two individual plants was 30 m to minimize sampling similar clones. Meanwhile, 30 soil samples at a depth of 30 cm were collected near the sampled plants at each site and then bulked by site for analyses of nitrogen (NO_3_-N), phosphorus, potassium, sulfur (SO_4_-S), and soil pH and electrical conductivity (E.C.) at ALS Laboratory Group (Saskatoon, Saskatchewan).

**Table 6:** Soil zone and rural municipality of 14 long-term grazing sites in Saskatchewan Canada used for alfalfa plant selection.

No	Population name	Soil zone	Rural municipality (RM)
1	Crooked River	Grey Wooded	RM 426
2	Shellbrook		RM 493
3	Erwood		RM 394
4	MacDowall	Black	RM 463
5	Duck Lake		RM 403
6	Rockhaven		RM 439
7	Arcola		RM 64
8	Dalmeny	Dark Brown	RM 344
9	Pike Lake		RM 345
10	Fillmore		RM 96
11	Ceylon	Brown	RM 39
12	Gull Lake		RM 139
13	Val Marie		RM 17
14	Moose Jaw		RM 45

### Reference cultivars for genetic diversity study

Ten random alfalfa plants from each of the 14 alfalfa populations from long-term grazing sites were selected for the genetic diversity study. To identify population structure of the 14 alfalfa to the Canadian commercial alfalfa cultivars released in western Canada from 1926 to 1980, 11 commercial alfalfa cultivars (Table 1S) were also included in the study. The seeds of the 11 alfalfa cultivars were germinated on top of a double layer of filter paper (VWR 413) in 9 cm plastic Petri-dishes. Then, 10 random germinated seeds per cultivar were transplanted into a 1/8-gallon pot (11 × 0.9 cm, Sun Gro Horticulture) filled with propagation soil mix containing 70–80% sphagnum peat moss, vermiculite and dolomite (SS#3, Sun Gro Horticulture Ltd.) at the College of Agriculture and Bioresources greenhouse at the University of Saskatchewan. The greenhouse has an 18-h photoperiod supplemented with high pressure sodium halogen lamps to a total of 490–550 μMs^−1^ m^−2^ PAR. The air temperature was 26 °C and 20 °C, for day/night, respectively. Relative humidity was maintained at 67%.

### Field experimental design

Four hundred twenty alfalfa plants collected from the 14 long-term grazing sites were propagated by stem cuttings. Then, the cloned plants were transplanted to a 1/4-gallon pot (13 × 12 cm, Sun Gro Horticulture) with propagation soil mix containing 70–80% sphagnum peat moss, vermiculite and dolomite (SS#3, Sun Gro Horticulture Ltd.) at the College of Agriculture and Bio-resources greenhouse in the University of Saskatchewan. A field spaced nursery was established on June 6, 2017, at the Agriculture and Agri-Food Canada (AAFC) Saskatoon Research Farm, Saskatoon, SK. The experimental design was a nested randomized complete block design (RCBD) with two blocks. There were 840 plots in the nursery (14 alfalfa populations × 30 genotypes population^−1^ × 2 blocks). Spacing between rows and plants within a row was 1 m. Weed control was done using a triple rototiller (KS-190, Tram sales Ltd, AB, Canada) in early spring in addition to hand weeding around individual plants. The soil was a Sutherland clay loam (Dark Brown Chernozem, Typic Haploloroll)^93^. Weather data for Saskatoon from 2018 to 2020 was obtained from Environment Canada (https://climate.weather.gc.ca). The average monthly air temperature and precipitation at the study site are shown in Fig. 3S. Growing season (May to September) air temperature during the three study years was near the long-term mean except for a higher temperature in May 2018, and lower temperatures in September 2018, May and August 2019. Rainfall was lower than normal during June and July in 2018. Almost no rainfall was recorded in May 2019. There was a higher than normal rainfall in June 2020.

### Agro-morphological trait measurement

Six agro-morphological traits were measured on individual genotypes for three consecutive summers from 2018 to 2020, in addition to nutritive value analysis in 2018 and 2019. Spring vigor (SV) was visually scored for each genotype using a 1–5 scale (poor to good) on the basis of spring growth, plant size and leafiness. Days to flower (GDD) was recorded as the date of first flower emerged on each genotype. Plant height (PH) for each genotype was measured from the soil surface to the tip of the stem by stretching the stems upwards. For forage dry matter yield (DMY) determination, each genotype was harvested, and dried at 60 °C for 48 h in a forced-air oven and was weighed in grams. For regrowth yield (RY) determination, each genotype was harvested, and dried at 60 °C for 48 h in a forced-air oven and was weighed in grams. Fall plant height (FPH) was measured from the soil surface to the tip of the stem by stretching the stems upwards for each genotype. The flowering date was expressed as growing degree days (GDDs) using a base temperature of 5 °C^94^.

### Forage nutritive value

For forage nutritive value determinations, plants at full bloom stage before the first cut were dried at 60 °C for 48 h in a forced-air oven. The dried plant samples were ground in a Wiley mill (Thomas-Wiley, Philadelphia, PA, USA) and then passed through a 1-mm mesh screen (Cyclone Mill, UDY Mfg., Fort Collins, Colorado, USA). Ground samples were stored in filter bags (Nasco Whirl–Pak, USA) prior to crude protein (CP), neutral detergent fiber (NDF) and acid detergent fiber (ADF) determinations. The values of three nutritive value traits were determined using a FOSS XDS rapid content analyzer (Foss, Denmark).

### Phenotypic data analysis

For each phenotypic trait, a linear mixed model was used to perform analysis of variance (ANOVA) using JMP (JMP 15.2.1. SAS Institute Inc., Cary, NC). Soil zone, year, population (soil zone) and genotype (population, soil zone), soil zone × year, were treated as fixed effects in the model. The block was treated as a random effect. For each of six agro-morphological and three nutritive values traits, if the ANOVA indicated a significant difference at *p* ≤ 0.05 level, means were separated using the studentized Tukey multi-treatment method. Degrees of freedom were calculated using Satterthwaite’s method.

Genotype-environment associations (GEA) were determined using the redundancy analysis (RDA) procedure using the R package “*vegan* 2.5-7 version” with the *rda* function^95,96^. The long-term (1986–2015) lowest and highest air temperatures and total precipitation (from May to September) for GEA analysis were obtained from Canada Weather Stats (available at https://www.weatherstats.ca/) and Environment Canada (available at https://climate.weather.gc.ca/). The soil pH, nitrogen (NO3-N), phosphorus (P), potassium (K), sulfur (SO4-S), and electrical conductivity (E.C.) from each long-term grazing site were also added into the GEA^40^. The electrical conductivity was removed as it was highly correlated ($\left| {\text{r}} \right|$ > 0.7) with soil pH, because the RDA method for GEA is a regression-based method subject to problems when using highly correlated predictors as described by Dormann et al.^97^. Two alfalfa populations from Dalmeny and Pike Lake were excluded from the GEA due to no soil test information.

Individual genetic data were considered as response matrix Y, and a set of environmental predictors were used as explanatory matrix X. Genotypes were coded using the values 0, 1 or 2 that correspond to homozygote for the most frequent alleles, and heterozygote and homozygote for the less frequent alleles. RDA aims at constructing a matrix Y′ of fitted genetic values estimated from the regression of each locus by the environmental predictors and at performing principal component analysis on the matrix Y′^98^. The number of axes used (*K*) is determined by looking at the amount of information retained on the different axes of the RDA. A Mahalanobis distance *D* is then computed for each locus to identify loci showing extreme *D* values compared to the rest of the SNPs. A Mahalanobis distance is a multidimensional generalization of the idea of measuring how many standard deviations is a point from an average point. Computation of the Mahalanobis distance uses the *covRob* function of R package “*ROBUST*”^99^. Mahalanobis distances are distributed as a chi-squared distribution with *K* degrees of freedom after correcting with the genomic inflation factor^100^. Inflation factors are constant values that are used to rescale chi-square statistics in order to limit inflation due to diverse confounding factors^101^. Inflation factors are constant values that are used to rescale chi-square statistics to limit inflation due to diverse confounding factors^101^. Multicollinearity between environmental predictors was assessed using the variance inflation (VIF) and since all predictors showed VIF < 5 none were excluded^102^. We then adjusted the resulting *p*-values for the false discovery rate (FDR) by computing *q*-values with the “*qvalue*” R package^103^. A locus is considered as an outlier if its *q*-value is < 0.01.

### DNA extraction and library establishment

Fresh young leaves of 10 random alfalfa genotypes from the 14 alfalfa populations from long-term grazing sites (except 11 genotypes for Shellbrook) and 10 genotypes from the 11 commercial alfalfa cultivars were collected into 1.5 ml centrifuge tubes and flash frozen in liquid nitrogen. Then the tubes were immediately stored in a − 80 $\mathrm{^\circ{\rm C} }$ freezer before the leaf samples were dried in a freeze-drier (Freezone 6, Labconco, USA) at − 52 °C and < 0.09 Pa for 72 h. One hundred milligram of dried leaf samples were ground individually with a sterilized plastic stick. The genomic DNA was extracted using DNeasy Plant Mini Kit (Qiagen, Toronto, ON, Canada). The Quant-iT PicoGreen dsDNA Assay Kit (Invitrogen, Carisbad, CA, USA) was used to quantify the concentration of the DNA samples. The DNA concentration was adjusted to 20 ng μl^−1^, and 100 ng DNA was digested by *ApeKI* (New England Biolabs, Ipswitch, MA). Then, the individual DNAs were ligated with a unique barcode adapter and a common adapter (Eurofins Genomics, Louisville, KY, USA) for 96 genotypes per plate using the protocol from Elshire et al.^25^. The ligated DNA fragments were purified by Qiagen PCR cleanup kit (Qiagen, Toronto, ON, Canada). Following the purification, PCR primers (Eurofins Genomics, Louisville, KY, USA) and KAPA HiFi HotStart Library Amp Kit (Roche, Laval, QC, Canada) were added through PCR amplification. The amplicon fragments were dual-side size selected using 0.57 × and 0.78 × (Average fragment size 400 bp, approximate size distribution 220–900 bp) using Sera Mag Select (Cytiva, Marlborough, MA, USA). An aliquot of each sample was run on an Agilent 2100 Bioanalyzer (Agilent Technologies 2013) for evaluation of fragment sizes. The three libraries of 96 barcoded samples were sequenced with paired-ends of 125 bp in three lanes using an Illumina HiSeq 2500 at the National Research Council of Canada, Saskatoon, Canada.

### SNP calling

The quality of pooled, raw reads was assessed using FastQC software^104^. The raw reads were then demultiplexed using Sabre^105^. After demultiplexing, the raw reads of individual samples were cleaned by removing adapters and low-quality sequences using Trimmomatic v.0.36 based on the default setting of paired-end mode, phred 33 and threads 6^106^. The *M. sativa* genome sequence^107^ was used as a reference genome for alignment of the reads using Burrows-Wheeler Aligner v0.7.17 software^108^. SNP calling was performed using Freebayes v1.2.0^109^ with the following settings: minimum coverage 1, minimum alternate fraction 0.1, minimum alternate count 5, genotype variant threshold 26, minimum base quality 13, pooled continuous, best 1 alleles, mapping quality 30, ploidy 2, other settings, default. The minor allele frequency, extent of missing SNP data and distribution of SNPs on the reference genome were calculated. The SNPs with Phred-scaled quality (QUAL) score ≥ 1260 were kept for imputation using BEAGLE software^110^. After imputation, 19,853 high quality SNPs were obtained and used in further analysis.

### Genetic diversity analysis

The Bayesian method implemented in the program STRUCTURE is a common method applied to detect population genetic structure^111–114^. This program uses a Bayesian clustering approach to define clusters containing groups of individuals whose genotypes maximize Hardy–Weinberg and linkage equilibrium^111^. A discriminant analysis of principal components (DAPC)^115^ using the “*adegenet*” package for R^116,117^ was conducted to identify clusters of genetically related individuals. The Bayesian Information Criterion (BIC) was used for selecting the optimal number of clusters (*K*) based on the elbow method^115^. The *find.clusters* function was used to detect the number of clusters among the populations. It uses K-means clustering which decomposes the total variance of a variable into between-group and within-group components. The optimum number of sub-populations has the lowest associated BIC. A cross validation function *Xval.dapc* was used to confirm the correct number of principal components (PC) to be retained.

The genetic diversity analysis was based on 19,853 SNP markers obtained from the SNP calling procedure. Three types of diversity analysis were performed at individual genotype level. First, genetic structure of 251 alfalfa genotypes was assessed using a model-based Bayesian method implemented in the program STRUCTURE version 2.3.4^111^. Sub-population numbers (*K*) ranging from 1 to 10 were evaluated, repeating each analysis 20 times. Modelling decisions included correlated allele frequencies and use of an admixture model. A burn-in of 30,000 iterations and 30,000 iterations of the Markov Chain Monte Carlo (MCMC) were used. The most likely number of sub-populations was inferred with the delta *K* method^118^ implemented in the STRUCTURE HARVESTER program^119^. The CLUMPP program^120^ was used to generate a consensus ancestry estimate from the 20 independent runs for each *K* and ancestry bar plots were generated with the “*pophelper*” package for R^117,121^.

### Identification of unique loci associated with long-term grazing

The 19,853 SNPs, with minor allele frequency (MAF) below 5% removed, were used for genotype-environment association for 122 genotypes representing 12 alfalfa populations sampled from the long-term grazing sites. The SnpEff software^122^ was used to annotate all SNPs based on the *M. sativa* reference genome database. The candidate genes were localized by retrieving the positions of significant SNP markers in the annotation database of *M. sativa* genome function^107^.

### Linkage disequilibrium

Pairwise Pearson correlation (r^2^) between pairs of SNPs were performed to assess the linkage disequilibrium (LD) across all chromosomes. A total of 12,402 SNPs were used in the LD analysis. The LD decay over physical distance was determined as the mean distance under the LD threshold of r^2^ = 0.2. The R package “*sommer*” (version 4.2.0) was used to conduct LD analysis^123^.

## Supplementary Information

Supplementary Figures.

Supplementary Tables.

## Data Availability

The datasets generated during the current study are available from Sequence Read Archive (SRA) database under NCBI [SRA accession PRJNA900199].
