# Supplementary material for: Genetic diversity and local adaption of alfalfa populations (Medicago sativa L.) under long-term grazing
Source: Sci Rep. 2023 Jan 30;13:1632. doi: 10.1038/s41598-023-28521-3 (PMC9886962; doi:10.1038/s41598-023-28521-3)
Supplement: Supplementary file 1 — Supplementary Figures. [file 41598_2023_28521_MOESM1_ESM.docx]

**Genetic Diversity and Local Adaption of Alfalfa Populations (*Medicago sativa* L.) under Long-Term Grazing**

Hu Wang^1^, Bruce Coulman^1^, Yuguang Bai^1^, Bunyamin Tarˈan^1^ & Bill Biligetu^1🖂^

^1^Department of Plant Sciences, College of Agriculture and Bioresources, University of Saskatchewan, Saskatoon, Saskatchewan, Canada. ^🖂^email: [Bill.Biligetu@usask.ca](mailto:Bill.Biligetu@usask.ca)

**Supporting information**


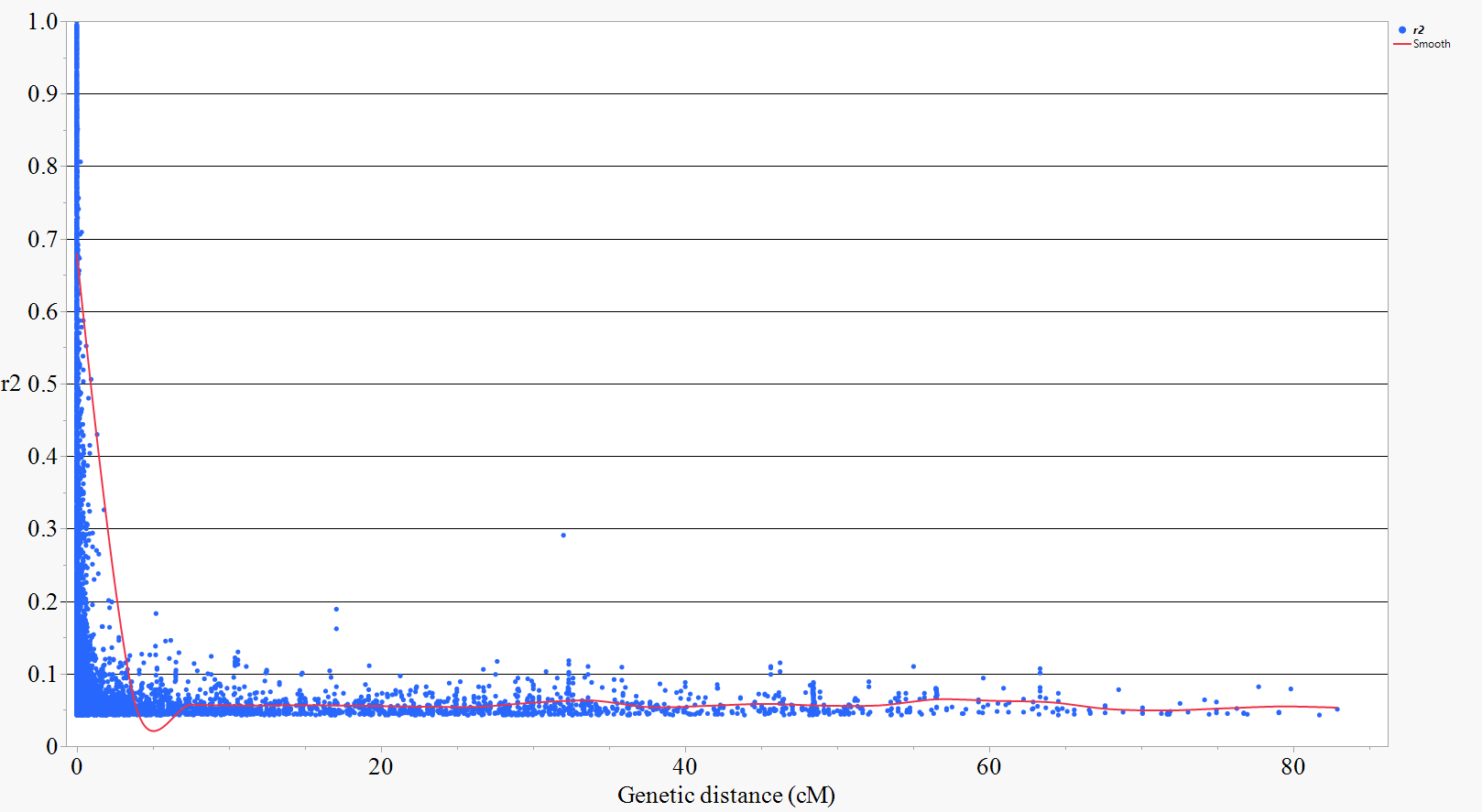
**Figure 1S.** A pairwise LD values (r^2^) are plotted against a genetic distance (cM). Inner fitted trend line is a nonlinear logarithmic regression curve of r^2^ on genetic distance.

**Figure 2S.** Linkage disequilibrium for SNPs within the same gene for (A) Winter extreme temperature; (B) soil sulfur; (C) Summer extreme temperature; and (D and E) soil potassium (K). Pairwise LD values (R^2^) were indicated by R^2^ color key legend bar.


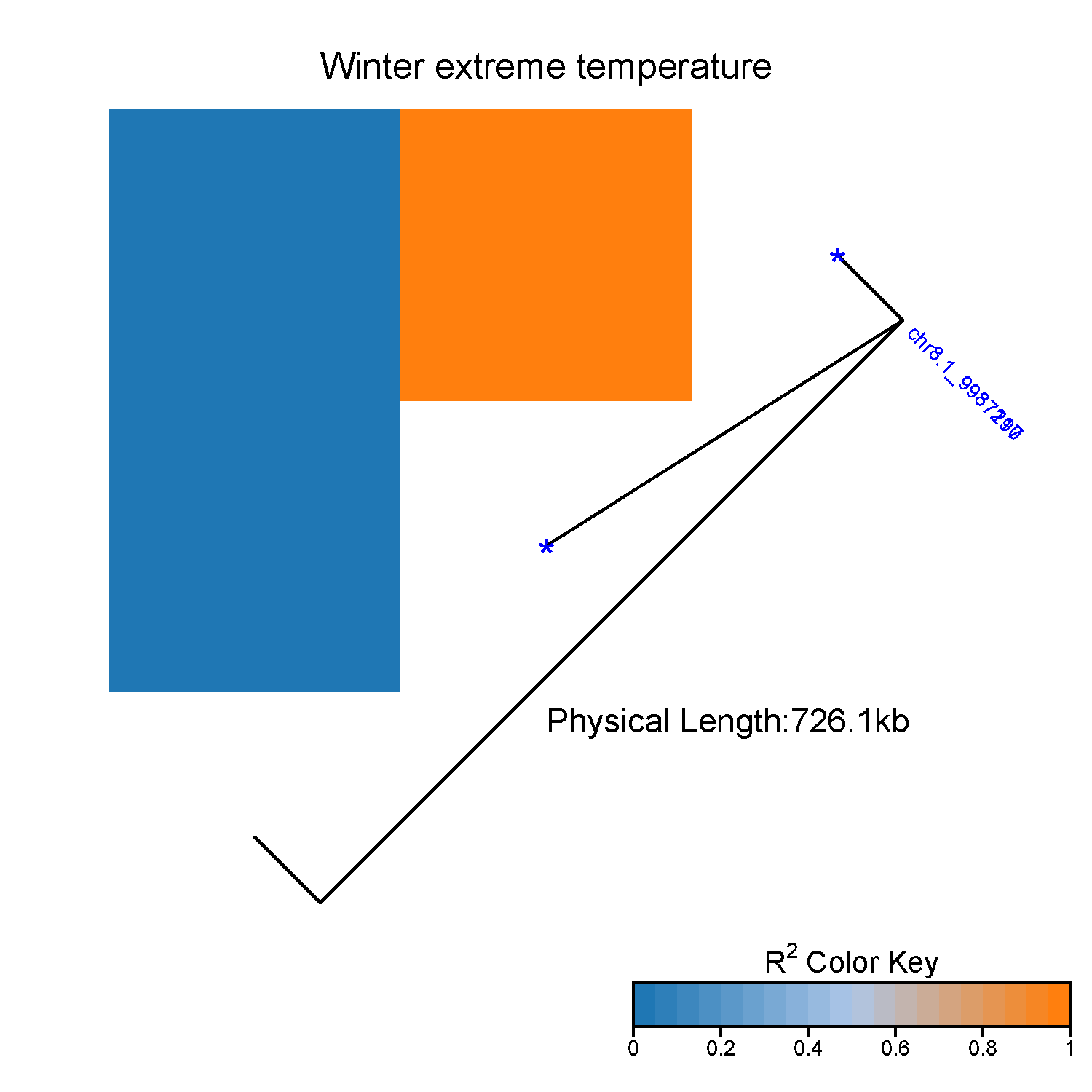

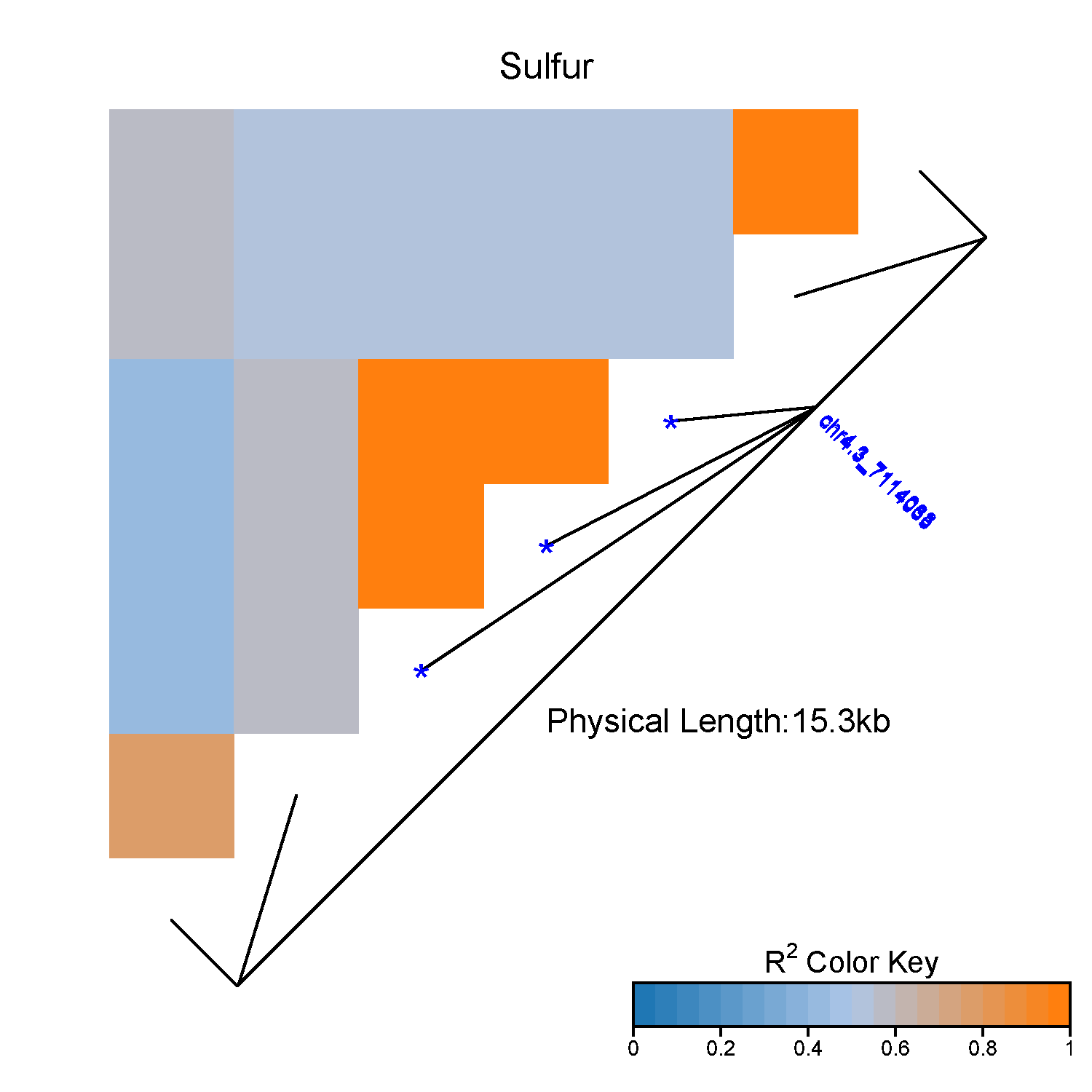

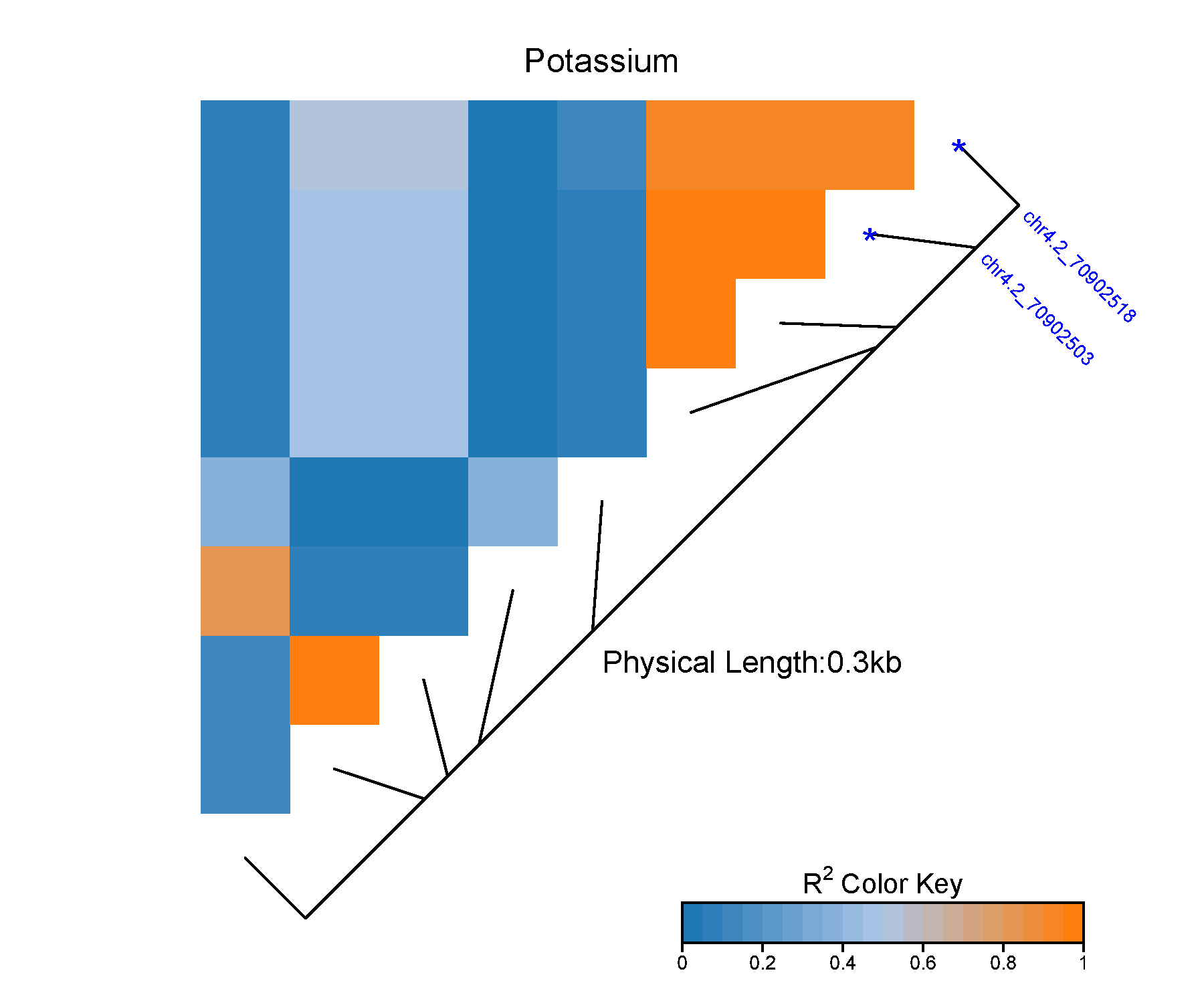

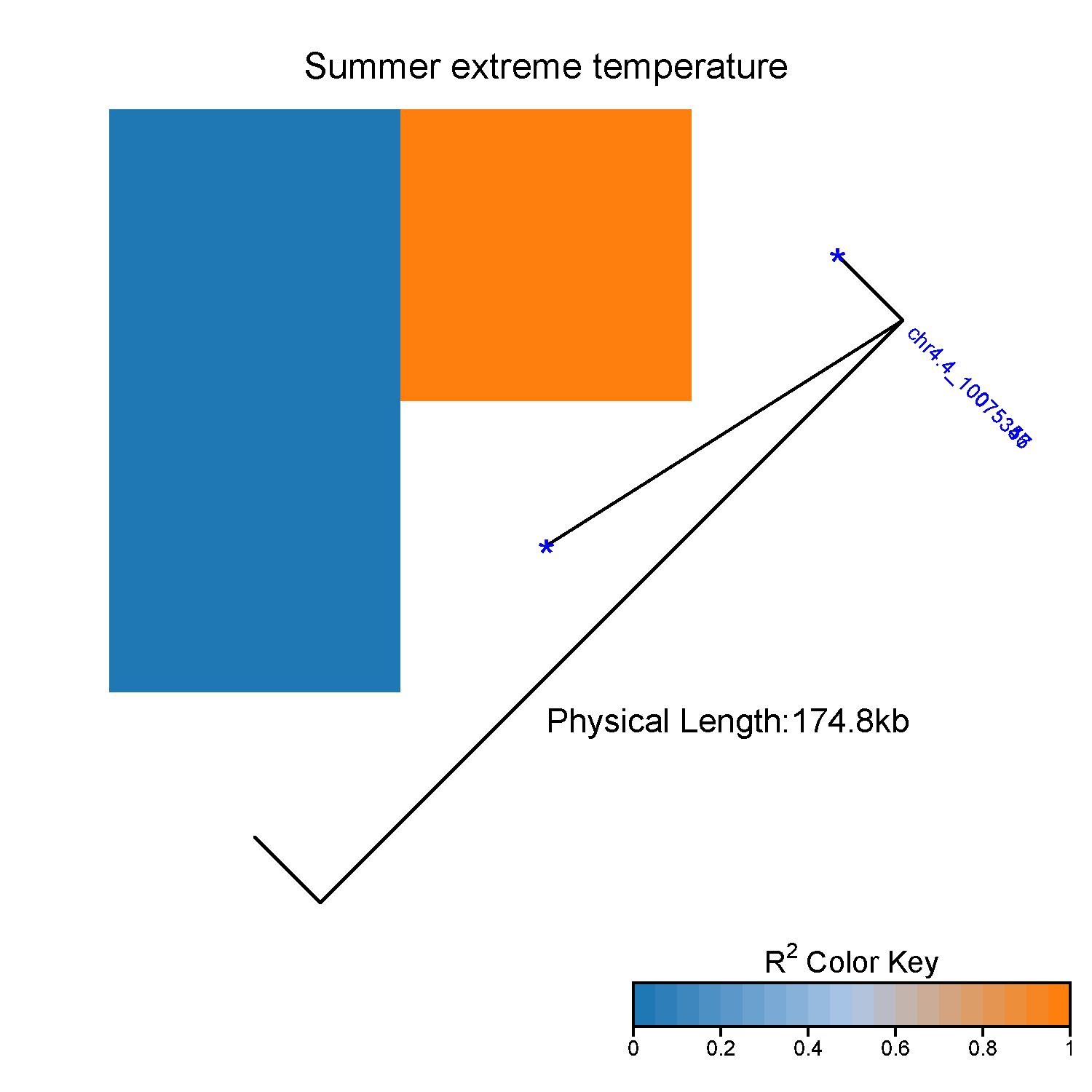

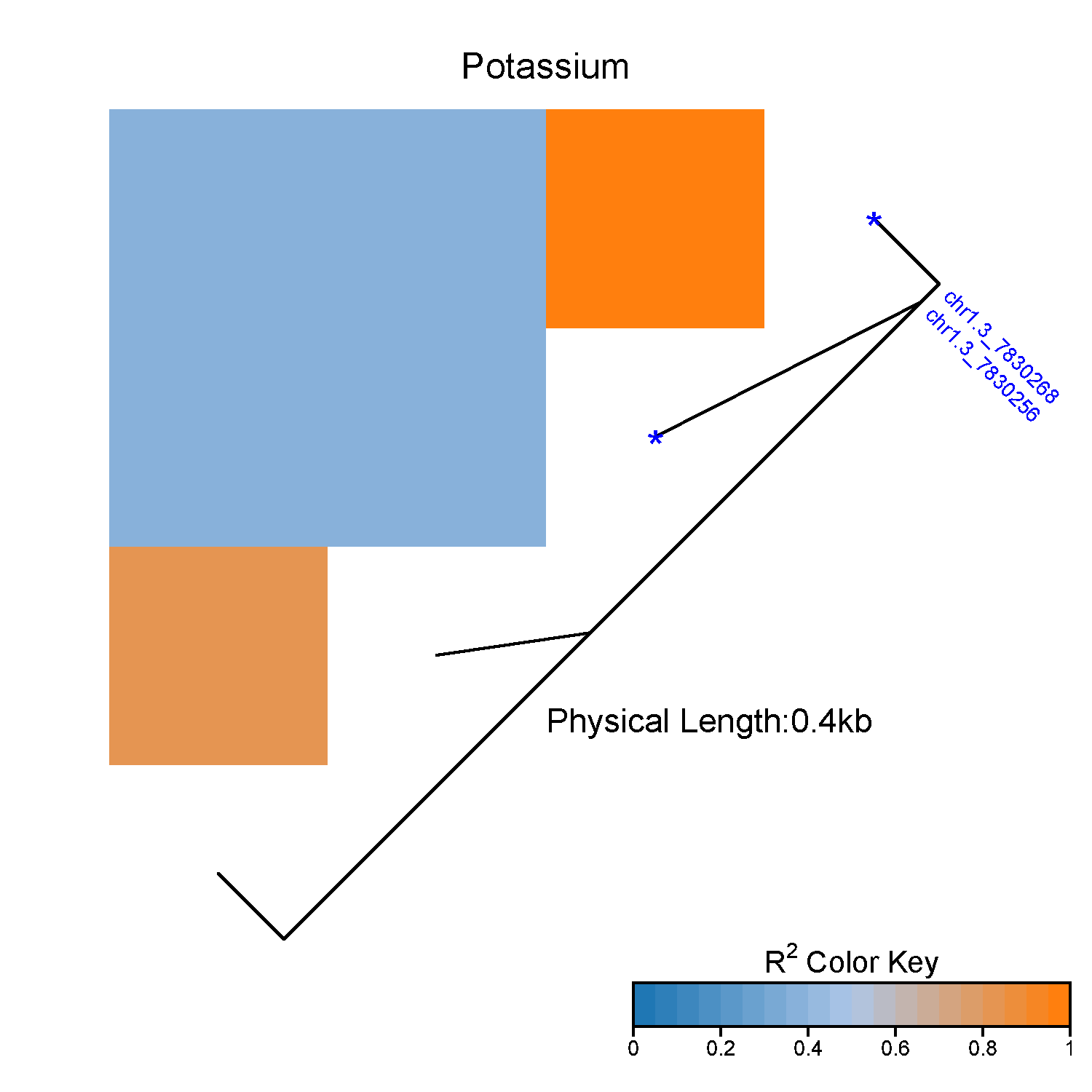


chr8.1_9987179

chr8.1_9987210

chr4.3_7114035

chr4.3_7114063

chr4.3_7114088

chr4.4_10075347

chr4.4_10075356

chr4.2_70902503

chr4.2_70902518

chr1.3_7830256

chr1.3_7830268

B

A

C

D

E


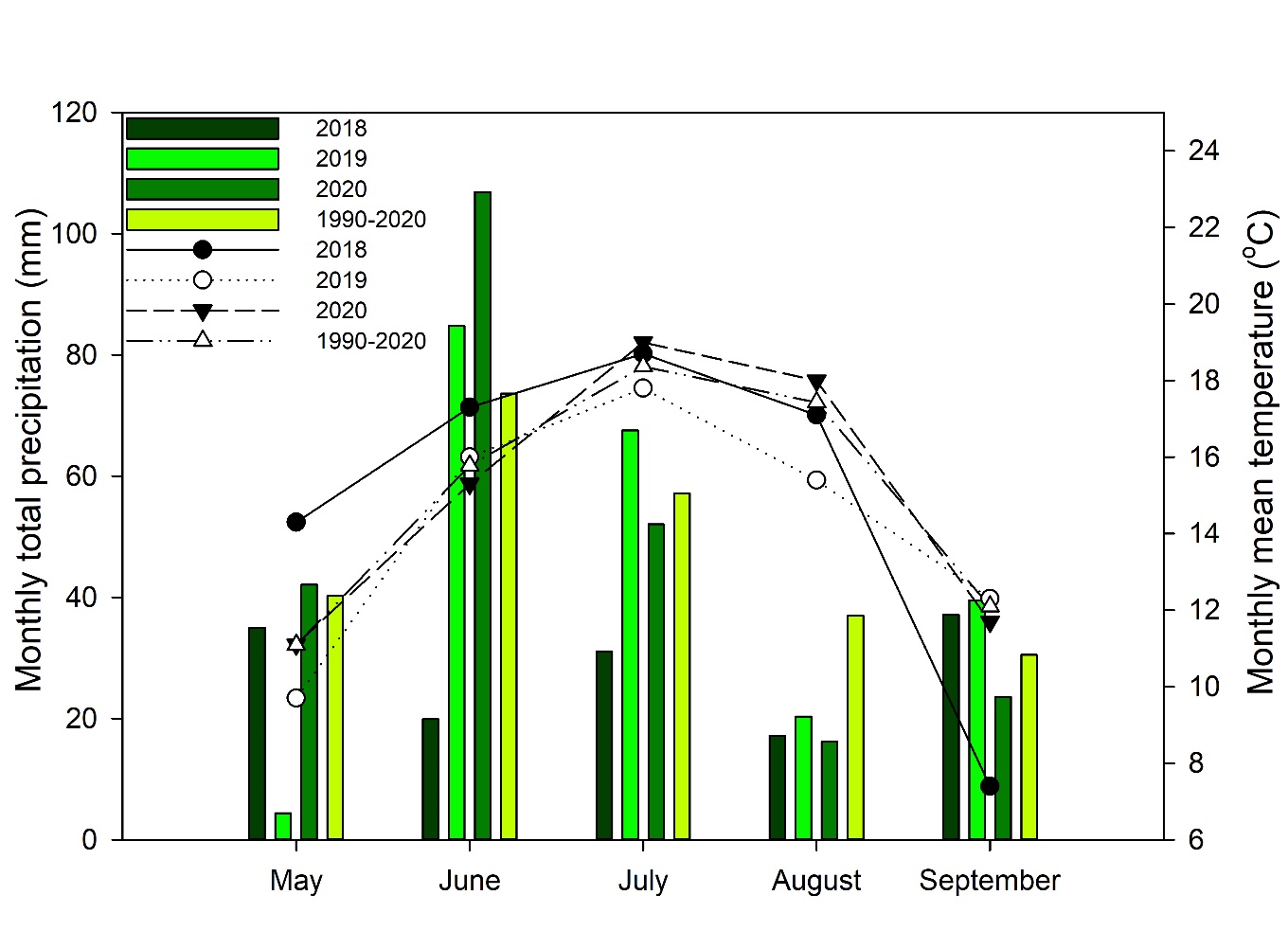
**Figure 3S.** Monthly mean air temperature (˚C) and monthly total precipitation (mm) during the growing seasons from 2018 to 2020 at Saskatoon, SK (Source: Environment Canada’s weather database).
