# Supplementary material for: Genetic diversity and local adaption of alfalfa populations (Medicago sativa L.) under long-term grazing
Source: Sci Rep. 2023 Jan 30;13:1632. doi: 10.1038/s41598-023-28521-3 (PMC9886962; doi:10.1038/s41598-023-28521-3)
Supplement: Supplementary file 2 — Supplementary Tables. [file 41598_2023_28521_MOESM2_ESM.docx]

**Genetic Diversity and Local Adaption of Alfalfa Populations (*Medicago sativa* L.) under Long-Term Grazing**

Hu Wang^1^, Bruce Coulman^1^, Yuguang Bai^1^, Bunyamin Tarˈan^1^ & Bill Biligetu^1🖂^

^1^Department of Plant Sciences, College of Agriculture and Bioresources, University of Saskatchewan, Saskatoon, Saskatchewan, Canada. ^🖂^email: [Bill.Biligetu@usask.ca](mailto:Bill.Biligetu@usask.ca)

**Supporting information**

**Table 1S.** The ancestry trace, fall dormancy level, flower color, country of origin and year of release of 11 commercial alfalfa cultivars seeded in Western Canada from 1926-1980.

| Cultivar | Country | Ancestry trace | Fall dormancy level | Flower color | Reference |
| --- | --- | --- | --- | --- | --- |
| Rangelander | Canada | Rambler (variegated), Roamer (variegated), Drylander (variegated) and *M. falcata* accession | 1 | Variegated | Heinrichs, et al. ^1^ |
| Anik | Canada | An accession of *M. falcata* from Russia | 1 | Yellow | Pankiw and Siemens ^2^ |
| Algonquin | Canada | *M. media* and Rhizoma (*media*) | 2 | Variegated light purple | Baenziger ^3^ |
| Anchor | USA | Apex and Alfa, Dupuits and Saranac | 3 | Purple | Barnes ^4^ |
| Drylander | Canada | *M. sativa*, *M. media* and *M. falcata* | 3 | Yellow | Heinrichs ^5^ |
| Roamer | Canada | Ladak (variegated), Cossack (*media*), Ranger (variegated), Rhizoma (*media*), Hardistan and Siberian (*falcata*) | 2 | Variegated | Heinrichs ^6^ |
| Beaver | Canada | Ladak, Turkestan (var. Turkestanica), Cossack (*media*), Viking, Grimm (variegated), Rhizoma, unnamed hardy strains from Universities of Nebraska and Wisconsin | 2 | Purple | Bolton, et al. ^7^ |
| Rambler | Canada | Siberian, Ladak (variegated) and Rhizoma (*media*) | 1 | Variegated | Heinrichs and Bolton ^8^ |
| Vernal | USA | Cossack, Ladak (variegated), *M. media* var. Kansas Common, yellow-flowered diploid *M. falcata* | 2 | Variegated | Hanson, et al. ^9^ |
| Ladak | India | *Falcata* type (other claimed as subsp. × varia), subject to natural selection in USA and Canada | 2 | Variegated | Garver ^10^ |
| Grimm | Canada | Strain 666 of ‘Grimm’ alfalfa (variegated), *M. media* | 2 | Variegated (predominantly purple and blue) | Hanson, et al. ^9^ |

**References cited in Table 1S**

1 Heinrichs, D. H., Lawrence, T. & McElgunn, J. D. Rangelander Alfalfa. *Canadian Journal of Plant Science* **59**, 491-492, doi:<https://doi.org/10.4141/cjps79-076> (1979).

2 Pankiw, P. & Siemens, B. Anik alfalfa. *Canadian Journal of Plant Science* **56**, 203-205, doi:<https://doi.org/10.4141/cjps76-031> (1976).

3 Baenziger, H. Algonquin alfalfa. *Canadian Journal of Plant Science* **55**, 1093-1094, doi:<https://doi.org/10.4141/cjps75-173> (1975).

4 Barnes, D. *Alfalfa germplasm in the United States: Genetic vulnerability, use, improvement, and maintenance*, <<https://handle.nal.usda.gov/10113/CAT78694267>> (1977).

5 Heinrichs, D. H. Drylander alfalfa. *Canadian Journal of Plant Science* **51**, 430-432, doi:<https://doi.org/10.4141/cjps71-084> (1971).

6 Heinrichs, D. Roamer alfalfa. *Canadian Journal of Plant Science* **47**, 220-221, doi:<https://doi.org/10.4141/cjps67-040> (1967).

7 Bolton, J. L., Peake, R. W. & Downey, R. K. Registration of beaver alfalfa^1^ (Reg. No. 22). *Crop Science* **5**, 483, doi:<https://doi.org/10.2135/cropsci1965.0011183X000500050038x> (1965).

8 Heinrichs, D. H. & Bolton, J. L. *Rambler Alfalfa*. Vol. Publication 1030 (1958).

9 Hanson, C. H., Garrison, C. S. & Graumann, H. O. *Alfalfa varieties in the United States*, <<https://books.google.ca/books?id=TjfcXSaiGLsC&printsec=frontcover&redir_esc=y#v=onepage&q&f=false>> (1960).

10 Garver, S. *Alfalfa in South Dakota: Twenty-one years of research at the Redfield station*, <<https://openprairie.sdstate.edu/agexperimentsta_bulletins/383/>> (1946).
